# Integrated omics data of two annual ryegrass (*Lolium multiflorum* L.) genotypes reveals core metabolic processes under drought stress

**DOI:** 10.1186/s12870-018-1239-z

**Published:** 2018-01-30

**Authors:** Ling Pan, Chen Meng, Jianping Wang, Xiao Ma, Xiaomei Fan, Zhongfu Yang, Meiliang Zhou, Xinquan Zhang

**Affiliations:** 1 Department of Grassland Science, College of Animal Science and Technology, Sichuan Agricultural University, Chengdu, China; 2 Chair of Proteomics and Bioanalytics, Technical University of Munich, 85354 Freising, Germany; 3 Agronomy Department, University of Florida, Gainesville, USA; 4 Vazyme Biotech Co., Ltd, Nanjing State Economy & Technology Development Zone, Red Maple Technology Industrial Park, Nanjing, China; 5 Institute of Crop Sciences, Chinese Academy of Agricultural Sciences, Beijing, China

**Keywords:** Metabolism, Drought, Annual ryegrass (*Lolium multiflorum*), Omics, Transcriptome, Proteome, Metabolome

## Abstract

**Background:**

Annual ryegrass (*Lolium multiflorum* L.) is a commercially important, widely distributed forage crop that is used in the production of hay and silage worldwide. Drought has been a severe environmental constraint in its production. Nevertheless, only a handful of studies have examined the impact of short-term drought stress on annual ryegrass. The aim of this study was to explore how stress-induced core metabolic processes enhance drought tolerance, or adaptation to drought, in annual ryegrass.

**Results:**

We profiled the transcriptomes, proteomes, and metabolomes of two annual ryegrass genotypes: the drought-resistant genotype “Abundant 10” and drought-susceptible genotype “Adrenalin 11.” We identified differentially expressed metabolites and their corresponding proteins and transcripts that are involved in 23 core metabolic processes, in response to short-term drought stress. Protein–gene–metabolite correlation networks were built to reveal the relationships between the expression of transcripts, proteins, and metabolites in drought-resistant annual ryegrass. Furthermore, integrated metabolic pathways were used to observe changes in enzymes corresponding with levels of amino acids, lipids, carbohydrate conjugates, nucleosides, alkaloids and their derivatives, and pyridines and their derivatives. The resulting omics data underscored the significance of 23 core metabolic processes on the enhancement of drought tolerance or adaptation to drought in annual ryegrass.

**Conclusions:**

The regulatory networks were inferred using MCoA and correlation analysis to reveal the relationships among the expression of transcripts, proteins, and metabolites that highlight the corresponding elements of these core metabolic pathways. Our results provide valuable insight into the molecular mechanisms of drought resistance, and represent a promising strategy toward the improvement of drought tolerance in annual ryegrass.

**Electronic supplementary material:**

The online version of this article (10.1186/s12870-018-1239-z) contains supplementary material, which is available to authorized users.

## Background

Drought is a severe environmental constraint to seed germination, plant growth, and productivity [1, 2]. Plants employ physiological and molecular mechanisms of drought tolerance to cope with water shortages. Indeed, the drought-response mechanisms developed by plants at the cellular level are essential, as they allow tolerance in plants that facilitates cellular homeostasis [3]. Mounting evidence has confirmed that plant species that are more tolerant to drought stress maintain higher levels of unsaturated fatty acids, such as hexadecenoic acid, palmitic acid, pimelic acid, stearic acid, and linolenic acid, all of which intervene in cases of compromised membrane fluidity and cellular functions [4–6]. In addition, the synthesis of amino acids, such as valine, leucine, phenylalanine, proline, and histidine, can contribute to turgor maintenance through osmotic adjustment when plants, especially resistant cultivars, are subjected to gradually increasing drought stress [7].

Plants exhibiting high drought tolerance are promising candidates for studies in drought-related genes, proteins, and metabolites [8]. Transcriptomic, proteomic, and metabolomic profiling are highly useful approaches to dissecting the complex networks of regulatory mechanisms at multiple levels in plants [9, 10]. In addition, multiple co-inertia analysis (MCoA) is a critical method for integrating data from several multi-omics datasets [11]. One of the advantages of MCoA is that it can be used to analyze a subset of variables (e.g., transcripts, proteins, and metabolites) that are present in two or more datasets. Several recent studies using comparative physiological, metabolomic, and transcriptomic analyses have provided deep insights into the mechanisms associated with improving abiotic stress resistance through the application of exogenous melatonin [12]. Similarly, combined analyses of the transcripts, proteome, and metabolites have been used to understand the manner in which core metabolic processes enhance carotenoid synthesis in transgenic maize [13]. “Omics” studies have also been performed, using large drought datasets for forage grasses with differing levels of sensitivity, both under water stress and non-stress conditions [14, 15].

Annual ryegrass (*Lolium multiflorum* L.), a species closely related to perennial ryegrass (*Lolium perenne*), is a commercially important forage crop that is widely cultivated for the production of hay and silage worldwide [16], including southern China. In recent years, severe short-term (daily to monthly) droughts have occurred frequently over southern China, causing a severe adverse effect on grass productivity [17, 18]. As such, the economic importance of annual ryegrass has encouraged many researchers to study the physiological and molecular bases of drought tolerance. Nevertheless, only a handful of studies have examined the impact of short-term drought stress on annual ryegrass, using an omics approach [19].

In our recently published study, we developed two annual ryegrass varieties, Abundant 10 and Adrenalin 11, with differing degrees of drought tolerance [20]. In order to explore the molecular mechanism of drought tolerance in these two annual ryegrass genotypes, we identified differentially expressed metabolites and their corresponding proteins and transcripts that are involved in 23 core metabolic processes under short-term drought treatment. The associated regulatory networks were inferred using MCoA and correlation analysis, to reveal the relationships among the expression of transcripts, proteins, and metabolites that highlight the corresponding elements of these core metabolic pathways. This study provides valuable insight into the molecular mechanisms of drought resistance and represents a promising approach toward the improvement of drought tolerance in annual ryegrass.

## Methods

### Plant samples and drought treatments

Two *L. multiflorum* genotypes, drought-resistant “Abundant 10” and drought-susceptible “Adrenalin 11,” were used in this study. Seedlings were transplanted 7 days after germination into Hoagland’s nutrient solution. All seedlings were grown in temperature-controlled growth chambers with 16-h photoperiods (25°/18 °C day/night temperature) and relative humidity of 60%. At day 20, after germination, the seedlings were divided into four groups to be used as the control (0 h) and various drought-treated samples. The drought-treated samples were placed on plastic trays and naturally air-dried for 1, 2, and 24 h, respectively (Additional file 1 Figure S1). Ten individual plants from each treatment group were considered a biological replicate. After treatment, three biological replicates were used for total RNA and protein extraction, and six biological replicates were then subjected to gas chromatography–mass spectrometry- (GC-MS)-based metabolite identification.

Relative water content (RWC) was calculated using the formula described by Barrs and Kozlowski [21]. The chlorophyll content was calculated according to the method described by Lakra et al. [22]. Relative electrical conductivity (REC) was calculated as the ratio of the initial electrical conductivity (EC) to the final EC [23]. The changes and activity of malondialdehyde (MDA) concentration, superoxide dismutase (SOD), catalase (CAT), and ascorbic acid peroxidase (APX) were each assayed with MDA, SOD, CAT, and APX assay kits, respectively (Comin Biotechnology Co. Ltd., Suzhou, China). A one-way ANOVA was performed using the SPSS Statistics 20.0 software (IBM Corp, Armonk, NY, USA), and means were compared using the least significant difference (LSD) test to determine significant differences (*P* < 0.05).

### Transcriptome sequencing and analysis

Total RNA was isolated from samples, using the TRIzol reagent (Agilent Technologies, Santa Clara, USA). A mass of 5 μg per sample was collected for cDNA library construction, using the NEB Next® Ultra™ Directional RNA Library Prep Kit for Illumina® (NEB, USA). The cDNA Library was validated by two different methods to determine the average molecular length: 1) the Agilent 2100 Bioanalyzer (Agilent DNA 1000 Reagents; Agilent Technologies) and 2) real-time quantitative PCR (QPCR; TaqMan Probe; Thermo Fisher Scientific, Waltham, MA, USA). The qualified libraries (average length of fragment was between 250 and 350 bp) were amplified on the cBot System to generate the cluster on the flow cell using the TruSeq PE Cluster Kit V3-cBot-HS (Illumina, San Diego, CA, USA). The amplified flow cell was subjected to paired-end sequencing using the HiSeq 2000 sequencing system (Illumina, USA). As a reference genome had not been previously created, the clean reads were assembled as the reference genome via the Trinity software (https://github.com/trinityrnaseq/trinityrnaseq/wiki) [24]. Read counts per gene were expressed as the expected number of fragments per kilobase of transcript sequence per million base pairs sequenced (FPKM). The *P*-values were adjusted using the Benjamini and Hochberg’s approach to control the false discovery rate (FDR). Gene Ontology (GO) enrichment analysis of differentially expressed genes (DEGs) was implemented with the GOseq R software package, in which the gene length bias was corrected. The GO terms with DEGs (FDR ≤ 0.001 and a fold change ≥2) were used for functional enrichment analysis. Genes with an adjusted *P*-value below 0.05, as determined by the DESeq software, were assigned as differentially expressed, and employed in the GO and Kyoto Encyclopedia of Genes and Genomes (KEGG) analyses. The KEGG enrichment analysis of DEGs in the KEGG database (http://www.genome.jp/kegg/) and the KOBAS (KEGG Orthology Based Annotation System) software [25, 26] were used to test the statistically significant enrichment of DEGs in KEGG pathways.

### Protein identification and data analysis

Proteins were extracted from samples at four time points throughout the drought stress treatments: 0, 1, 2, and 24 h. The extractions were performed with Lysis Buffer 3 containing 1 mM phenylmethylsulfonyl fluoride (PMSF) and 2 mM ethylenediaminetetraacetic acid (EDTA), and suspended at 200 W for 15 min. Proteins were isolated by centrifugation at 30000 *g* for 15 min at 4 °C, after which 5× volume of chilled acetone and 10% (*v*/v) trichloroacetic acid were added at − 20 °C.

After two rounds of centrifugation, the supernatant was carefully discarded and the precipitate was washed three times with cold acetone. The protein pellet was air-dried by lyophilization and dissolved in Lysis Buffer (7 M urea, 2 M thiourea, 4% NP40, 20 mM Tris-HCl, pH 8.0–8.5). The protein pellet was then suspended for 15 min and centrifuged at 4 °C at 25000 *g* for 15 min, and the supernatant was collected.

To reduce the number of disulfide bonds in the proteins of the supernatant, 10 mM dithiothreitol (DTT) was added, and the mixture was left for 1 h at 56 °C. Subsequently, 55 mM iodoacetamide (IAM) was added to block the cysteines, after which samples were kept in a dark room for 1 h. The supernatant of the proteins was kept at − 80 °C. Protein samples of 100 μg each were added to 2.5 μg Trypsin (Promega, Madison, WI, USA) with a weight ratio of 40 protein: 1 trypsin, and kept at 37 °C for 4 h. The peptides were vacuum-dried using Strata-X, and reconstituted in 0.5 M triethylammonium bicarbonate (TEAB) based on the manufacturer’s protocol for the 8-plex iTRAQ reagent (Applied Biosystems, USA). This solution contained one unit of thawed and reconstituted iTRAQ reagent in 24 μL isopropanol. The peptides were labeled with isobaric tags, pooled, and then vacuum-dried.

Each fraction was re-suspended in buffer A (2% acetonitrile [ACN], 0.1% formic acid [FA]) and centrifuged at 20000 *g* for 10 min. The final concentration of peptides was on average approximately 0.5 μg/μL. The supernatant (10 μL) was evaluated using a LC-20 AD Nano-HPLC pump (Shimadzu, Kyoto, Japan) with an autosampler, and peptides were eluted onto an analytical C18 column (inner diameter 75 μm and column length 15 cm). The samples were loaded over 4 min, and the solvent gradient was run from 5% buffer B (96% ACN, 0.1% FA) for 0–8 min, with a linear gradient to 35% buffer B for 8–43 min, maintained at 60% buffer B for 43–48 min, and returned to 5% buffer B for 55–65 min.

Data acquisition was performed using a Triple TOF 5600 System (SCIEX, Framingham, MA, USA) fitted with a Nanospray III source (SCIEX) and a pulled quartz tip as the emitter (New Objectives, Woburn, MA, USA). Data were acquired using an ion spray with a 2.5 kV voltage. The curtain and nebulizer gases were set at 30 psi and 15 psi, respectively, and the interface heater temperature was 150 °C. The mass spectrometer was operated with a resolving power (RP) of 30,000 FWHM (full width at half maxima) for time-of-flight mass spectrometry (TOF/MS) scans. Survey scans were obtained in 250 ms and up to 30 product ion scans were acquired (cut-off threshold was 120 counts/s). Raw data files were transformed into Mascot generic format (MGF) files using the Proteome Discoverer software.

Raw data files acquired from the Orbitrap analyzer were converted into MGF files using the Proteome Discoverer 1.2 software (Thermo Fisher). The Mascot 2.3.02 search engine (Matrix Science, London, UK) was used to identify and quantify proteins. An automatic decoy database search was performed in Mascot, by choosing the decoy checkbox in which a random sequence database was generated and tested for raw spectra. The real database was also tested for raw spectra. Only peptides with a 95% confidence interval from the Mascot probability analysis were counted as identified. The identification of each protein involved at least one unique peptide. The quantitative protein ratios were weighted and normalized using the median ratio determined by Mascot. We considered only data with values of *P* < 0.05 and fold changes > 1.2 as significant. Functional annotations of identified proteins were performed using the Blast2GO program against the non-redundant (NR) protein database. The KEGG and clusters of orthologous groups (COG) databases were applied to classify the identified proteins.

### Metabolome analysis

Six biological replicates of each sample were analyzed for non-treated and drought-treated seedlings (drought for 24 h) in the two *L. multiflorum* genotypes. Approximately 50 mg of powdered samples were extracted in 1 mL of an 80% methanol, 20% distilled water solution for 30 min at 4 °C. During the extraction process, the samples were centrifuged for 10 min at 12,000 *g*. The supernatant was removed and the pellet was subjected to further extraction in 60% methanol, and then in water at 4 °C, as described above. Metabolite profiling was performed on an Agilent 1290 Infinity Liquid Chromatography System (Agilent Technologies) equipped with a 2.1 mm × 100 mm C18 reverse-phase column, with a 1.8-μm particle size (Waters Corp., Milford, MA, USA). The column was maintained at 40 °C and the injected sample volume was 4 μL. Mass spectrometry (MS) experiments were performed on an Agilent 6530 Accurate-Mass Q-TOF/MS (Agilent Technologies) equipped with an electrospray ionization source. The binary gradient had a flow rate of 0.35 mL/min, and a gradient elution from 5% to 95% acetonitrile separated all compounds. The total run time was 34 min.

The electrospray source parameters were optimized as follows: positive mode- sampling cone 35 kV, capillary voltage 4 kV, extraction cone 3 V, source temperature 100 °C, desolvation temperature 350 °C, cone gas flow 50 L/h, and desolvation gas flow 600 L/h. Negative mode- sampling cone 50 kV, capillary voltage 3.5 kV, extraction cone 4 V, source temperature 100 °C, desolvation temperature 300 °C, cone gas flow 50 L/h, and desolvation gas flow 700 L/h. For accurate mass acquisition, a lock mass of leucine enkephalin (Lock mass) at a concentration of 0.2 ng/mL was used via a lock spray interface for the positive ion mode ([M + H] + = 556.2771 Da) and negative ion mode ([M − H] − = 554.2615 Da) to ensure accuracy during the MS analysis.

After filtration, a peak table was created that included information on the retention time (RT), mass, and ion intensity of all identified components. Raw data were first preprocessed with a Mass Profiler (Agilent Technologies) and input to the Simca-P 13.0 program for multivariate analysis (i.e., principal component analyses [PCA] and partial least squares discriminant analysis [PLS-DA]). Data were expressed as the mean ± SD. Variable importance in the projection (VIP) values were obtained for variables in the orthogonal projections to latent structures discriminant analysis (OPLS-DA) model in order to select the differentially expressed metabolites. An independent *t*-test (*P* < 0.05) was used to determine whether the individual metabolites (candidate biomarkers) obtained from PLS-DA modeling, exhibited statistically significant differences between groups at the univariate analysis level. Only the metabolites with a VIP > 1 and a *P*-value < 0.05 were defined as differential metabolites. The MS analysis system was used to identify the characteristic metabolites corresponding to the featured peak in the Metlin database (http://metlin.scripps.edu) [27]. The KEGG database was also used to link differential metabolite levels to functional metabolic pathways in the drought-treated groups, compared to those in the control groups.

### Integrative analysis using multiple co-inertia analysis and gene set enrichment analysis

The MCoA was performed using the Bioconductor software package omicade4 [28]. For consistency, we selected 1-, 2-, and 24-h treatments as the drought treatment groups, and 0 h as the control group. The 0-h and 24-h metabolomic profiles were used as the control and drought treatment profiles, respectively. The mean FPKM in the transcriptomic data (three replicates), ratios in the proteomics data (two replicates), and peak intensity in the metabolic data (six replicates) were used in the MCoA. All values were log-transformed. The MCoA can deal with multiple datasets; therefore, we did not merge the positive and negative channels in the metabolite profiling, which resulted in four datasets for each of genotypes. The Gene Set Enrichment Analysis (GSEA) software (version 2.2.2) was used for gene set enrichment analysis [29]. The gene set information was collected from GO and KEGG pathway databases, as in the differential expression analysis. Gene sets containing more than 1000 or fewer than five candidates were excluded. A permutation text with 1000 permutations was used to evaluate the significance of the enrichment results. The default settings for other parameters were used.

### Quantitative real-time-PCR analysis and western blot

Changes in the expression of 26 genes were verified by real-time PCR analysis, and detailed information on these genes is listed in Additional file 2 Table S1. Real-time RT-PCR was performed on the ABI7500 Real-Time PCR System (Applied Biosystems) using the SYBR® Premix EX-Taq™ II kit. Reverse transcription was performed using the Bio-Rad iScript cDNA Synthesis Kit. The PCR amplifications were conducted in a volume of 20 μL, containing 10 μL PCR-mix, 2.5 μL of genomic DNA, 5.5 μL ddH2O, and 1 μL of each primer. The thermocycler was set to touchdown mode according to the following program: 95 °C for 30 s, 1 cycle; 95 °C for 5 s, 40 cycles; and 60 °C for 34 s, 1 cycle. A melting curve was generated by heating the sample to 95 °C. Real-time PCR data was then analyzed by the comparative CT method.

An equal amount (10 μg) of each protein sample was loaded for sodium dodecyl sulfate polyacrylamide gel electrophoresis (SDS-PAGE) (1.5 mm gel thickness). Migration of proteins in the gel was conducted at 150 V until the blue band from the sample buffer ran out of the gel. Protein-Marker IV was also loaded to determine the molecular weight of the proteins. Proteins were then transferred onto a polyvinylidene fluoride (PVDF) membrane (Millipore, USA). The following antibodies were used in the western blot analysis: actin (ACT), chitinase (CHN), glutamine synthetase (GS1), methionine synthase (MTR), UDP-glucose pyrophosphorylase (UGPase), glutamate dehydrogenase 1 (GDH1), serine hydroxyl methyltransferase (SHMT), and glutamine synthetase (GlnA) (Agrisera, Sweden). The PVDF membrane was probed with primary antibodies and developed using enhanced chemiluminescence detection (PerkinElmer, Waltham MA, USA). The blots were detected using the BeyoECL Plus (P0018) system. The images were obtained using the ChemiDoc TM MP imaging system, and the quantifications were conducted with the Image Lab TM V5.1 software.

## Results

### Drought stress induced growth and physiological changes in *L. multiflorum*

**Fig. 1:**
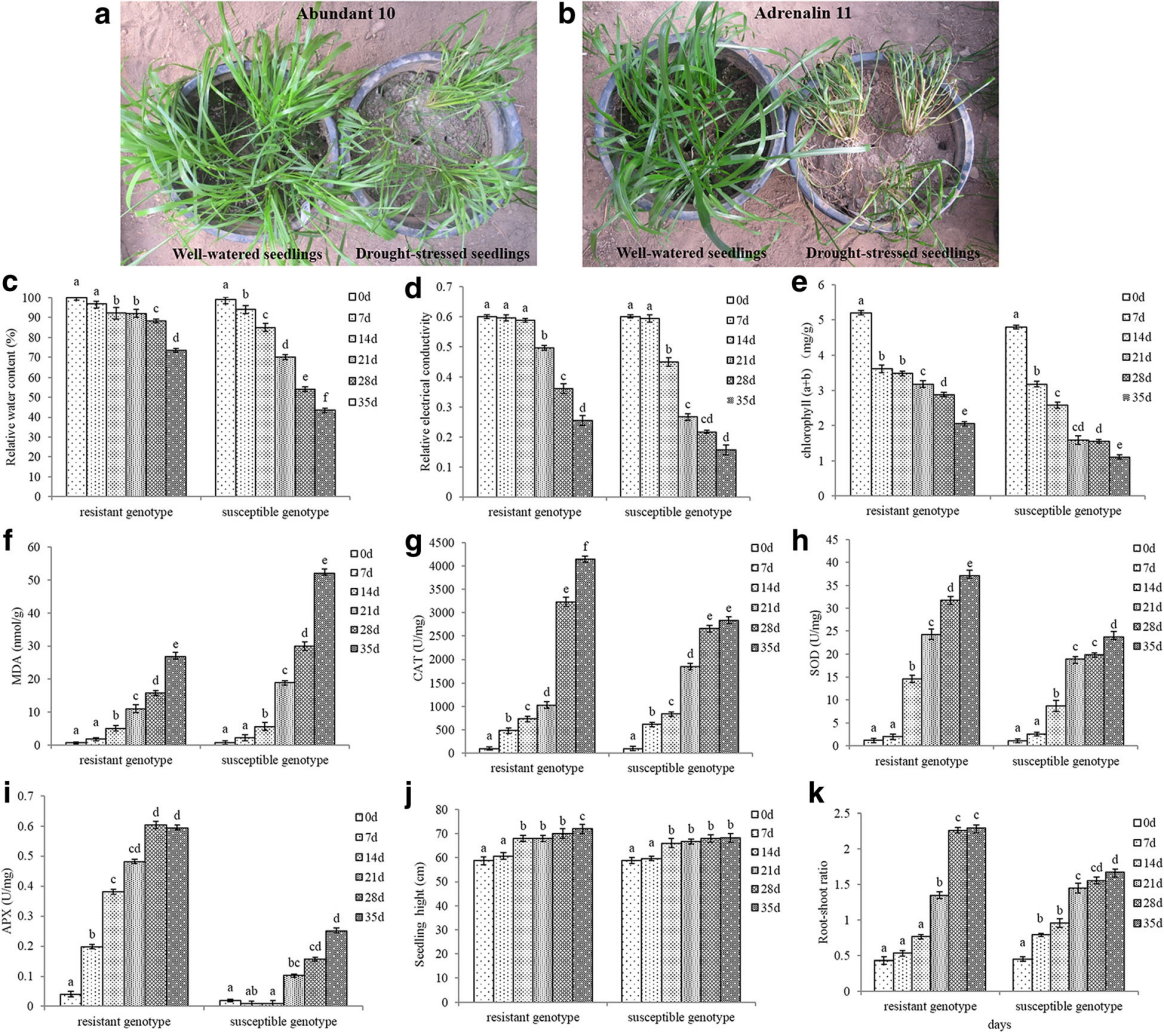
Representative images of two *L. multiflorum* genotypes under long term drought stress for 5 weeks (**a** and **b**). Morphological and physiological characters of annual ryegrass were measured after drought stress for 7 to 35 days (**c**–**k**). Different letters above bars indicate significant differences (*P* < 0.05) between different time points

To determine the morphological and physiological impact of drought stress on annual ryegrass, both drought-resistant and drought-susceptible genotypes were subjected to sustained drought for 5 weeks (Fig. 1a–b). Differences between the genotypes were observed in seedling height (SH), chlorophyll (a + b) content, REC, RWC, root-to-shoot ratio (RSR), malondialdehyde (MDA) content, catalase (CAT), superoxide dismutase (SOD), and ascorbic acid peroxidase (APX) activities (Fig. 1c–k). The impact of drought stress on RWC was apparently reduced among treated seedlings after the 14-day drought treatment, and there was a significant difference in response between the two annual ryegrass genotypes (Fig. 1c). The REC and chlorophyll (a + b) content of the susceptible plants exhibited a dramatic reduction, in comparison to those of the tolerant plants (Fig. 1d–e). Prior to the application of drought stress, MDA content was invariant among the control samples of the two annual ryegrass genotypes. However, susceptible genotype revealed significantly higher MDA contents, relative to those of the tolerant genotype after 14 days of drought stress (Fig. 1f). Higher levels of CAT, SOD, and APX activity were observed among the tolerant genotype exposed to long-term drought (Fig. 1g–i), whereas no significant changes were noted in SH when subjected to drought stress (Fig. 1j). However, a highly significant increase in RSR was observed in the tolerant genotype in comparison to the susceptible genotype after annual ryegrass seedlings were treated with drought stress for 21 days (Fig. 1k).

### Metabolite profiling of two *L. multiflorum* genotypes revealed changes in metabolites under drought stress

The differentially expressed metabolites of the two *L. multiflorum* genotypes were identified following exposure to 24 h of drought stress, using LC-MS and the OPLS-DA model. Different types of compounds were detected in positive and negative modes including: lipids; amino acids; organic acids; carbohydrates and carbohydrate conjugates; nucleosides, nucleotides, and their analogs; indoles and their derivatives; alkaloids and their derivatives; amine compounds, pyridines and their derivatives, among others (Additional file 3: Figure S2). additional(DEMs) exhibited contrasting expression levels between the drought-tolerant and drought-sensitive genotypes under drought stress, particularly lipids, amino acids, organic acids, amine compounds, and pyridines and their derivatives. Significant correlations were noted among the levels of these compounds, according to Spearman two-tailed correlation analyses (*P* ≤ 0.05 and *r*^2^ > 0.65 or *P* ≤ 0.05 and *r*^2^ < − 0.65; Additional file 4: Figure S3). Lipids and amino acids more favorably ionized in positive ionization mode (Additional file 4: Figure S3A), whereas organic acids generated higher signal intensities in positive ionization mode (Additional file 4: Figure S3). We found that lipid levels exhibited the greatest number of significant correlations with other metabolites, followed by amino acids, and organic acids.

Among these compounds, a portion of the DEM scan was mapped onto multiple core metabolic processes, for example, valine, leucine, and isoleucine biosynthesis; valine, leucine, and isoleucine degradation; α-linolenic acid metabolism; biotin metabolism; phenylalanine, tyrosine, and tryptophan biosynthesis; tropane, piperidine, and pyridine alkaloid biosynthesis; phenylalanine metabolism; sphingolipid metabolism; fatty acid biosynthesis; phenylpropanoid biosynthesis; glycerolipid metabolism; pantothenate and CoA biosynthesis; histidine metabolism; tryptophan metabolism; galactose metabolism; purine metabolism; cysteine and methionine metabolism; fatty acid metabolism; arginine and proline metabolism; 2-oxocarboxylic acid metabolism; cutin; suberin and wax biosynthesis; biosynthesis of amino acids; and the citrate cycle (TCA cycle; Table 1).

**Table 1:** Differentially expressed metabolites mapped to KEGG metabolic pathways

Metabolite Name	HMDB ID	KEGGCompound ID	Fold change (sus_24 vs.res_24)	Pathway Name
Pheophorbide a	METPA1634	C18021	0.911	Porphyrin and chlorophyll metabolism
Hexahomomethionine	METPA1758	C17233	−1.056168721	2-Oxocarboxylic acid metabolism
Colnelenic acid	HMDB30996	C16320	0.688	alpha-Linolenic acid metabolism
Traumatic acid	HMDB00933	C16308	0.665	alpha-Linolenic acid metabolism
Stearidonic acid	HMDB06547	C16300	−0.965724523	alpha-Linolenic acid metabolism
Caffeyl alcohol	METPA1708	C12206	0.689	Phenylpropanoid biosynthesis
Phytosphingosine	HMDB04610	C12144	−0.608	Sphingolipid metabolism
Abietinal	HMDB34735	C11887	−0.680	Diterpenoid biosynthesis
Picolinic acid	HMDB02243	C10164	−1.009229646	Tryptophan metabolism
Palmitoleic acid	HMDB03229	C08362	−1.552	Fatty acid biosynthesis
Alpha-Linolenic acid	HMDB01388	C06427	0.672	alpha-Linolenic acid metabolism
Guanosine 2’,3’-cyclic phosphate	HMDB11629	C06194	1.852	Purine metabolism
Hydroxyphenylacetylglycine	HMDB00735	C05596	2.027	Tyrosine metabolism
Galactosylglycerol	HMDB06790	C05401	1.183	Galactose metabolism Glycerolipid metabolism
2-Methoxyestradiol	HMDB00405	C05302	−0.991682395	alpha-Linolenic acid metabolism
13(S)-HPOT	METPA0543	C04785	−1.989038788	alpha-Linolenic acid metabolism
Imidazoleacetic acid ribotide	HMDB06032	C04437	0.681	Histidine metabolism
5-Methylthioribose	HMDB01087	C03089	1.626	Cysteine and methionine metabolism
Pimelic acid	HMDB00857	C02656	0.753	Biotin metabolism
Capric acid	HMDB00511	C01571	0.835	Fatty acid biosynthesis
Syringin	METPA1704	C01533	0.968	Phenylpropanoid biosynthesis
7,8-Diaminononanoate	METPA0113	C01037	−0.961929908	Biotin metabolism
Porphobilinogen	HMDB00245	C00931	2.258	Porphyrin and chlorophyll metabolism
Sphinganine	HMDB00269	C00836	−1.600936161	Sphingolipid metabolism
LPA(0:0/18:2(9Z,12Z))	HMDB07852	C00416	−0.704	Glycerolipid metabolism Glycerophospholipid metabolism
Guanosine	HMDB00133	C00387	1.841	Purine metabolism
Xanthine	HMDB00292	C00385	2.484	Purine metabolism
Geranyl-PP	HMDB01285	C00341	0.759	Terpenoid backbone biosynthesis
Indoleacrylic acid	HMDB00734	C00331	2.158484941	Tryptophan metabolism
Palmitic acid	HMDB00220	C00249	−1.552	Fatty acid biosynthesis Fatty acid degradation Cutin, suberine and wax biosynthesis Fatty acid metabolism
L-Valine	HMDB00883	C00183	−1.016522341	2-Oxocarboxylic acid metabolism Biosynthesis of amino acids Valine, leucine and isoleucine degradation Valine, leucine and isoleucine biosynthesis Pantothenate and CoA biosynthesis Aminoacyl-tRNA biosynthesis
Proline	HMDB00162	C00148	−1.551840689	Biosynthesis of amino acids Aminoacyl-tRNA biosynthesis Arginine and proline metabolism
L-Histidine	HMDB00177	C00135	−0.993	Histidine metabolism Aminoacyl-tRNA biosynthesis Biosynthesis of amino acids
L-Leucine	HMDB00687	C00123	−2.034	2-Oxocarboxylic acid metabolism Biosynthesis of amino acids Valine, leucine and isoleucine degradation Valine, leucine and isoleucine biosynthesis Aminoacyl-tRNA biosynthesis
Anthranilic acid	HMDB01123	C00108	1.346	Biosynthesis of amino acids Tryptophan metabolism Phenylalanine, tyrosine and tryptophan biosynthesis
Phenylalanine	HMDB00159	C00079	−1.004301822	Phenylalanine metabolismPhenylalanine, tyrosine and tryptophan biosynthesis Phenylpropanoid biosynthesis Tropane, piperidine and pyridine alkaloid biosynthesis Aminoacyl-tRNA biosynthesis 2-Oxocarboxylic acid metabolism Biosynthesis of amino acids
Pyridoxal 5’-phosphate	HMDB01491	C00018	1.153	Thiamine metabolism Vitamin B6 metabolism

### Comparative proteomics and transcriptomic profiling reveals differences in the expression of proteins and genes regulating core metabolism

**Fig. 2:**
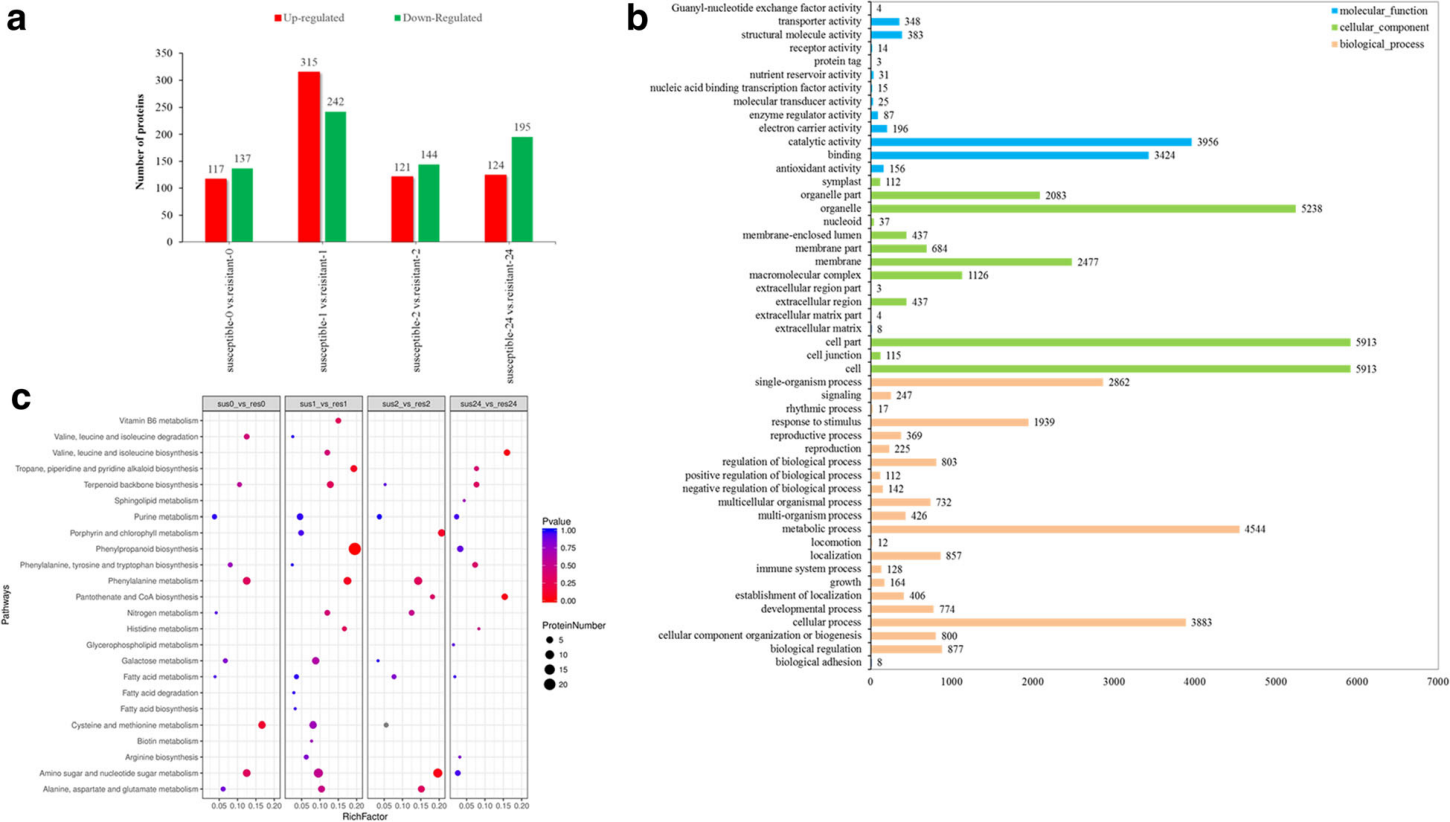
Comparison of upregulated and downregulated proteins in resistant and susceptible genotypes (**a**); Differentially expressed proteins (DEPs) were identified by a Gene Ontology (GO) analysis (**b**); DEPs involved in metabolic pathways related to metabolites identified by LC-MS were dramatically affected by drought in annual ryegrass (0, 1, 2, and 24 h) (**c**)

To investigate the differentially abundant proteins induced by drought treatment, we performed comparative proteomics analysis on the two annual ryegrass genotypes at four different time points 0, 1, 2, and 24 h. A total of 26,189 unique peptides matching 8224 proteins were identified by Mascot with a high level of confidence (all with a 1% FDR), of which 1395 were differentially abundant between the drought-susceptible and drought-resistant genotypes (Fig. 2a). To understand further the function and features of these proteins, differentially expressed proteins (DEPs) were identified using Blast2GO and the KEGG database. Most of these proteins were located in organelles, cells, membranes, or nuclei (Fig. 2b**)**. The KEGG pathway enrichment analysis revealed that the abundance of DEPs involved in metabolic pathways that are related to the metabolites identified by LC-MS were dramatically affected by drought (Fig. 2c). This identified the drought-induced proteins associated with stress responses that might contribute to enhanced tolerance or adaptation to drought in annual ryegrass.

**Fig. 3:**
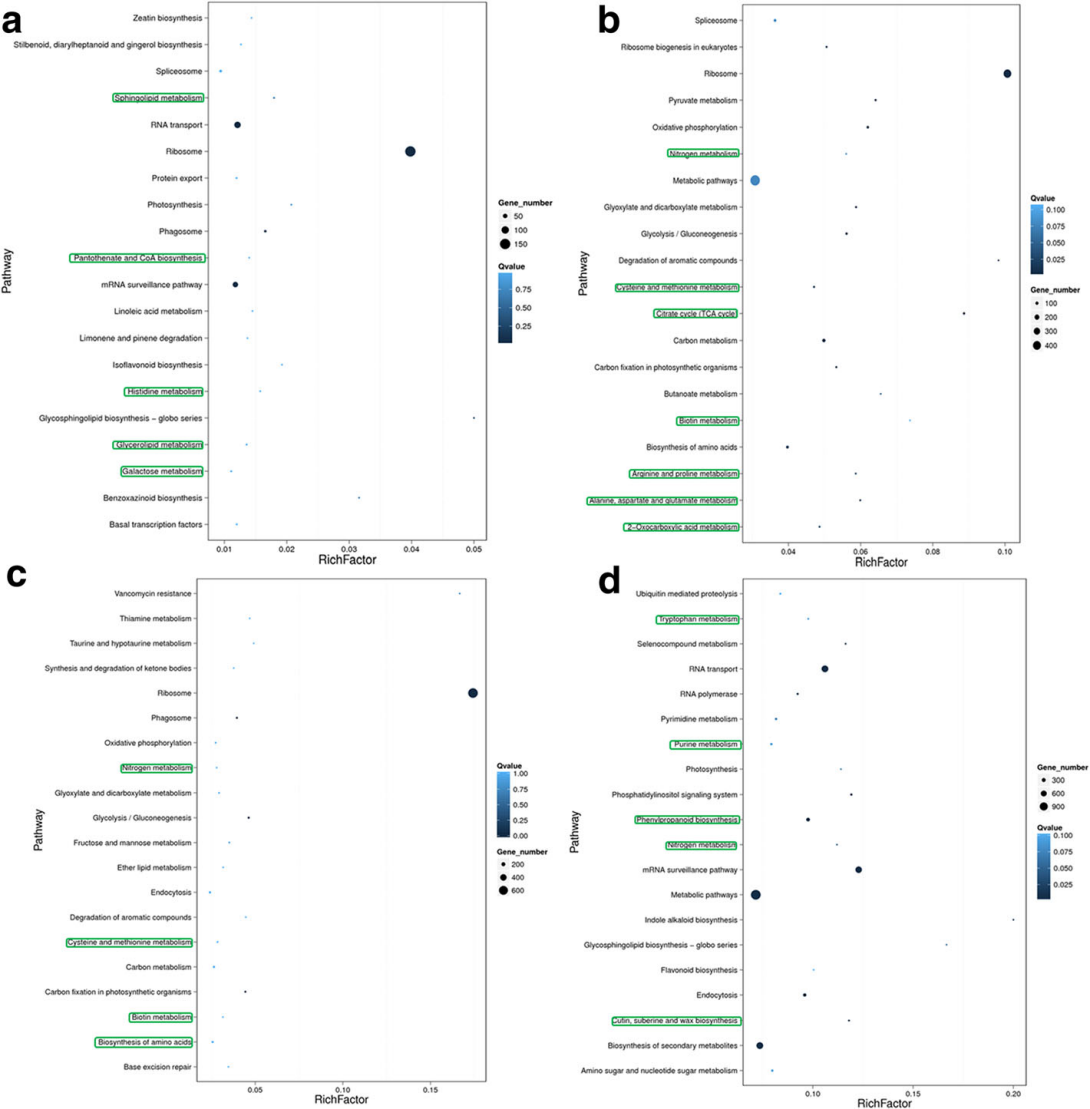
Differentially expressed genes involved in core metabolic pathways in two *L.multiflorum* genotypes at four time points (0, 1, 2, and 24 h) during drought treatment (**a**–**d**)

To gain insight into gene expression in annual ryegrass across all four time points of the drought treatment, we performed transcriptomic analysis to identify drought-mediated genes. After filtering out contaminated and low-quality Illumina HiSeq sequencing reads, approximately 137,708 unigenes were assembled. These annotated unigenes were used to search various functional databases, and unigenes that encoded transcription factors (TFs) were classified into different TF families (Additional file 5: Figure S4). Using a fold change > 2 and FDR < 0.05 as thresholds, transcripts from both resistant and susceptible genotypes were identified as DEGs. Many of the upregulated and downregulated DEGs that were highly expressed following drought treatment in the two *L. multiflorum* genotypes at different time points (Additional file 6: Figure S5), were enriched in various core metabolic pathways. Interestingly, a large number of DEGs that were involved in the pathway terms “sphingolipid metabolism,” “pantothenate and CoA biosynthesis,” “histidine metabolism,” “glycerolipid metabolism,” and “galactose metabolism,” were observed in both *L. multiflorum* genotypes before drought treatment (Fig. 3a). In comparison to the drought-susceptible genotype, the expression of many DEGs were significantly increased in tolerant genotype after 1 h of drought, including those associated with “nitrogen metabolism,” “cysteine and methionine metabolism,” “citric acid cycle (TCA cycle),” “biotin metabolism,” “arginine and proline metabolism,” and “2-oxocarboxylic acid metabolism” (Fig. 3b). Similarly, more than 100 genes involved in “nitrogen metabolism,” “cysteine and methionine metabolism,” “biotin metabolism,” and “biosynthesis of amino acids” were significantly enriched in tolerant genotype subjected to drought for 2 h (Fig. 3c). The number of DEGs involved in “tryptophan metabolism,” “purine metabolism,” “nitrogen metabolism,” and “cutin, suberine, and wax biosynthesis” was increased in tolerant genotype, compared to sensitive genotype after 24 h of drought stress (Fig. 3d). These results indicate that the number of genes involved in these core metabolic processes showed significant changes.

To further confirm the RNA-Seq data, qRT-PCR was performed. The expression trends of 26 amino acid and lipid metabolism-related genes were consistent with RNA-Seq data in the two *L. multiflorum* genotypes (Additional file 7: Figure S6), and fold changes in gene expression were significantly correlated in both the resistant and susceptible genotypes (r^2^ > 0.9).

### Multiple co-inertia analysis to evaluate the integration of omics datasets

**Fig. 4:**
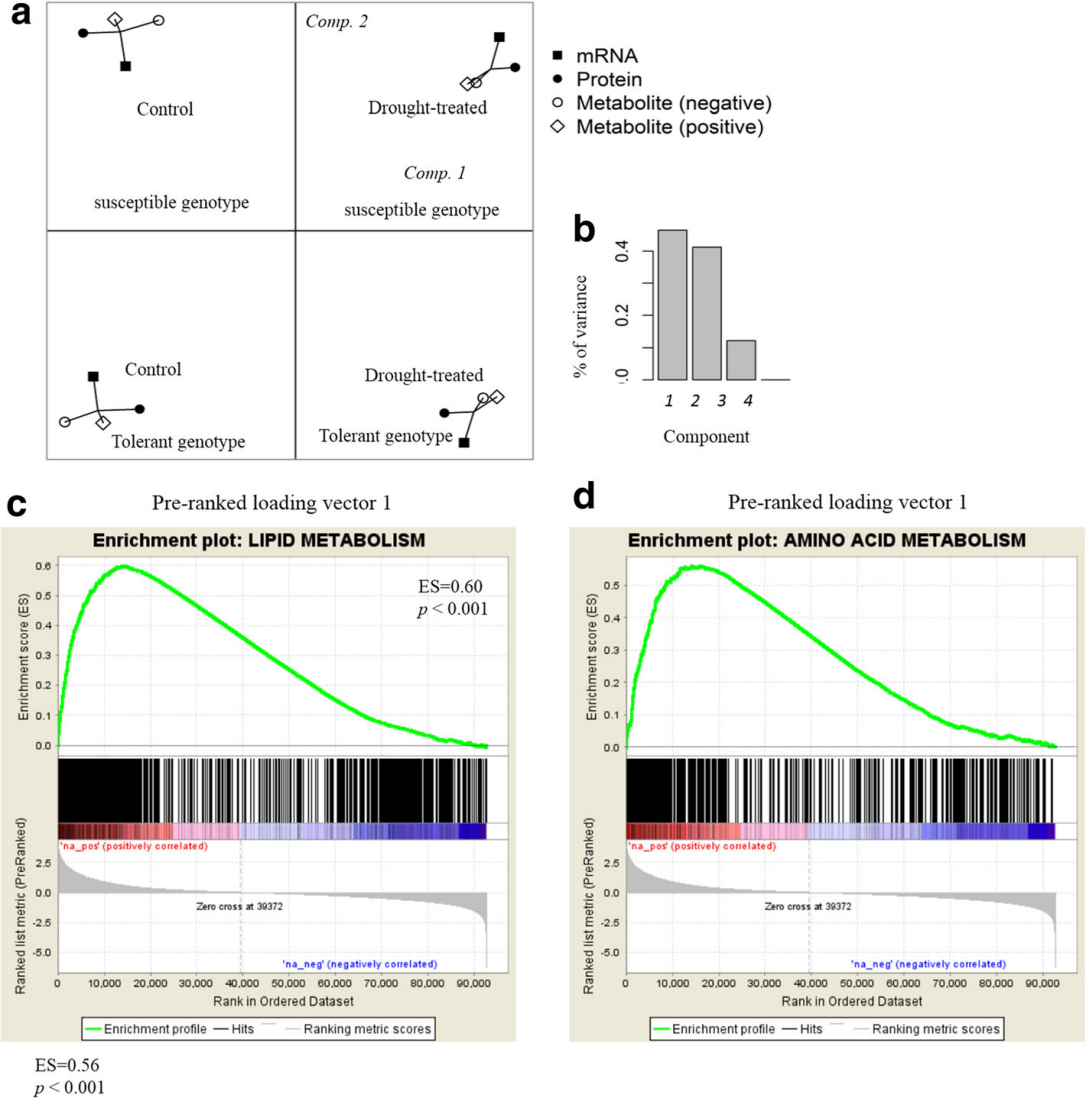
Integrative analysis of multiple omics data using MCoA and GSEA. The first two components defined by MCoA (**a**). The proportion of variation explained by each component (**b**). The GSEA of the first loading vector for the lipid metabolism (**c**) and amino acid metabolism gene sets (**d**). Each dot represents a plant genotype; the same genotype from different datasets are linked by segments. The length of segments connecting annual ryegrass is correlated with the similarity among datasets

To demonstrate the relationships among transcripts, proteins, and metabolites, we collapsed our samples into control and drought-treated seedlings using the means of each omics profile. A strong correlation between the four datasets was observed (Fig. 4a). The top two MCoA components, responsible for more than 80% of the total variation (Fig. 4b), clearly separated the drought stress sensitive and resistant genotypes (Fig. 4a) The first and second components accounted for roughly comparable proportions of the variation, implying that both genetic background and drought treatment underlie considerable variation in the data.

As in the PCA, in which the contributions of variables (e.g., transcript, protein, and metabolite levels) to a component could have been evaluated by their loading vectors, variables with either positive or negative associations (i.e., with high absolute values) had significant influence on the co-inertia components. In contrast to the PCA, the variables comprising MCoA loading vectors were derived from all four datasets. In consideration of the biological meaning of the MCoA components, we subjected the loading vectors of the two components to GSEA. Pre-ranked analysis was performed, in which variables were ranked according to their loadings. The loading vectors for component 1 were significantly and positively associated with amino acid metabolism and lipid metabolism (*P* < 0.001; Fig. 4c–d). Taken together, the omics data sets subjected to MCoA indicated that amino acid and lipid metabolism play important roles in annual ryegrass drought response.

### Targeted analysis revealed a significant association among transcripts, proteins, and metabolites in core metabolic processes

**Fig. 5:**
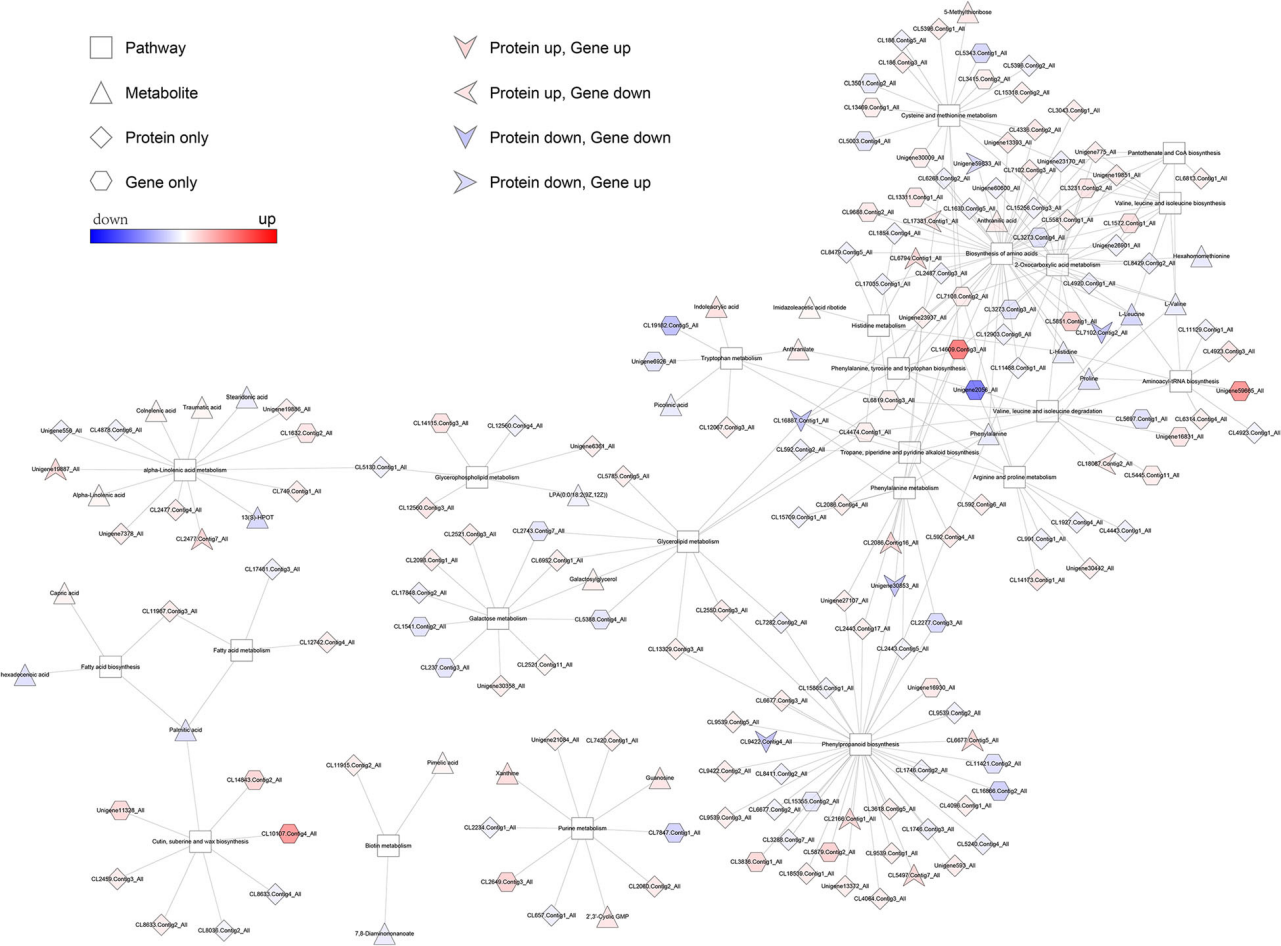
Trends in changes in transcripts and their corresponding proteins and metabolites, displaying the core metabolic processes involved in the response to drought in annual ryegrass

Correlation analysis was performed among differentially expressed metabolites, proteins, and genes (Fig. 5). We first matched all transcripts with their detected proteins, and an intermediate Pearson correlation coefficient of *r* = 0.724 was observed. The DEGs with corresponding DEPs were classified into different groups exhibiting upregulation or downregulation. A total of 47 DEGs exhibited trends that matched those of their corresponding proteins in the two annual ryegrass genotypes subjected to drought stress. These down-regulated DEG and DEP pairs were involved in “phenylalanine, tyrosine, and tryptophan biosynthesis,” “tropane, piperidine, and pyridine alkaloid biosynthesis,” “phenylalanine metabolism,” “phenylpropanoid biosynthesis,” “biosynthesis of amino acids,” “2-oxocarboxylic acid metabolism,” and “glycerolipid metabolism.” Similarly, 51 DEGs had contrasting expression changes with their corresponding proteins under drought stress, including genes involved in “valine, leucine, and isoleucine degradation” and “biosynthesis of amino acids.” In addition, a large number of transcripts or proteins that had no significant regulation were also observed in core metabolic pathways.

**Fig. 6:**
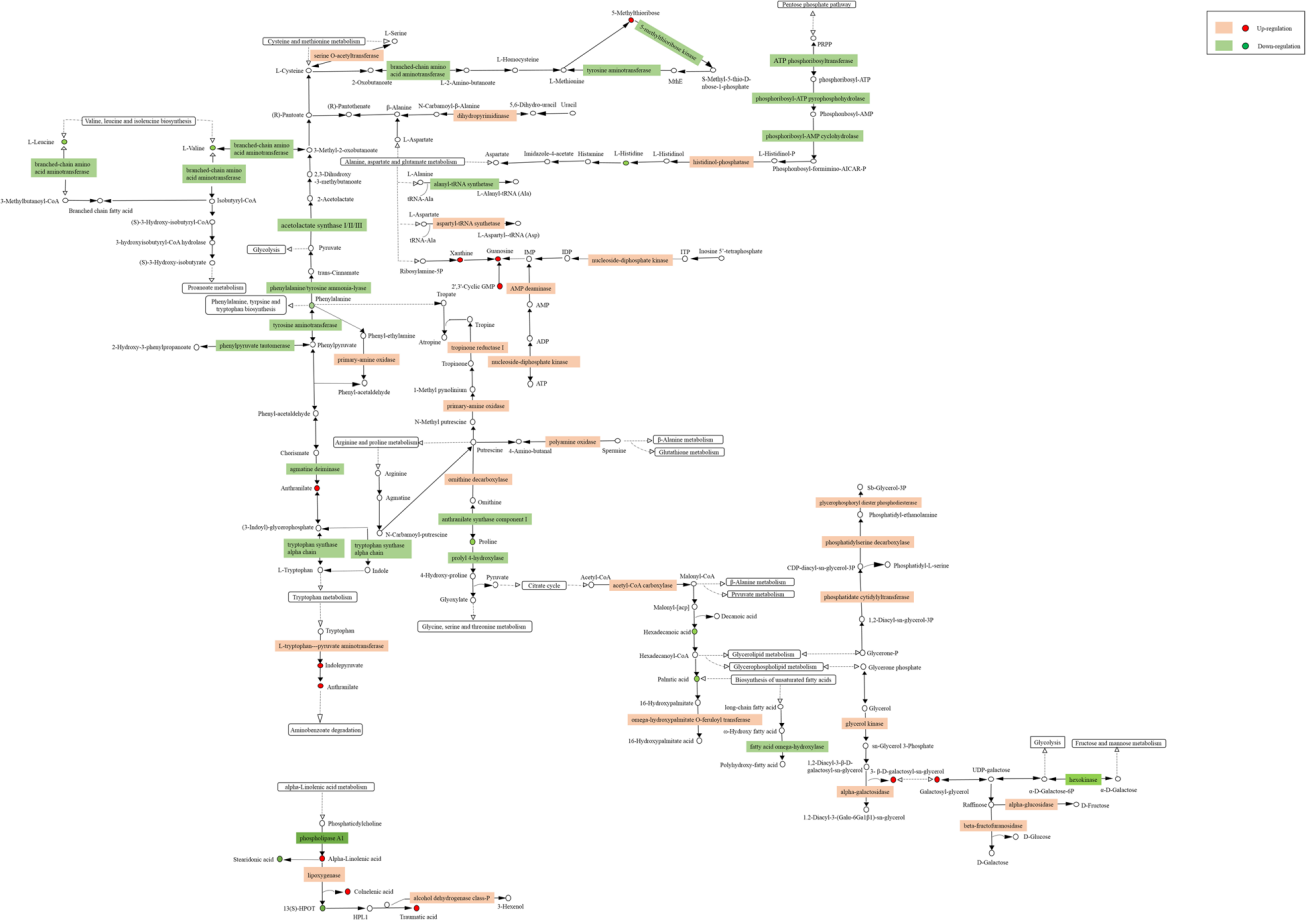
Establishment of correlation networks for the identification of regulatory mechanisms in drought-resistant *L. multiflorum*

Protein–gene–metabolite correlation networks were generated to examine responses of the 23 metabolic pathways in drought-resistant annual ryegrass (Table 2; Fig. 6). Integrated metabolic pathways were used to observe changes in enzymes, corresponding to levels of amino acids, lipids, carbohydrate conjugates, nucleosides, alkaloids and their derivatives, and pyridines and their derivatives. Several amino acids, such as L-valine, L-leucine, phenylalanine, L-histidine, and proline, were found in lower concentrations in the drought-susceptible genotype than in the tolerant genotype under drought conditions. This may be a consequence of the downregulated key enzymes, including branched-chain amino acid aminotransferase (EC 2.6.1.42), tyrosine aminotransferase (EC 2.6.1.5), 5-methylthioribose kinase (EC 2.7.1.100), ATP phosphoribosyl transferase (EC 2.4.2.17), phosphoribosyl-AMP cyclohydrolase (EC 3.5.4.19), phosphoribosyl-ATP pyrophosphohydrolase (EC 3.6.1.31), phenylpyruvate tautomerase (EC 5.3.2.1), agmatine deiminase (EC 3.5.3.12), prolyl 4-hydroxylase (EC 1.14.11.2), and the tryptophan synthase alpha chain (EC 4.2.1.20).

**Table 2:** Differentially expressed proteins and transcripts related to the core metabolism after drought in resistant and susceptible genotypes

Access ion	Description, EC number	Protein ratio (sus_vs.res_)	Gene ratio (sus_ vs.res_)
Unigene26901_All	acetolactate synthase I/II/III large subunit [EC:2.2.1.6]	+ 1.27	NA
Unigene775_All	acetolactate synthase I/III small subunit [EC:2.2.1.6]	+ 1.35	NA
Unigene23170_All	branched-chain amino acid aminotransferase [EC:2.6.1.42]	−0.81	NA
Unigene775_All	acetolactate synthase I/III small subunit [EC:2.2.1.6]	+ 1.35	NA
CL2443.Contig5_All	phenylalanine/tyrosine ammonia-lyase [EC:4.3.1.25]	−0.54	NA
CL16887.Contig1_All	tyrosine aminotransferase [EC:2.6.1.5]	−0.77	−0.77
CL2086.Contig4_All	primary-amine oxidase [EC:1.4.3.21]	+ 1.28	NA
CL2086.Contig16_All	primary-amine oxidase [EC:1.4.3.21]	+ 1.28	+ 9.18
CL15709.Contig1_All	phenylpyruvatetautomerase [EC:5.3.2.1]	−0.77	NA
CL991.Contig1_All	agmatinedeiminase [EC:3.5.3.12]	−0.77	NA
CL17035.Contig1_All	tryptophan synthase alpha chain [EC:4.2.1.20]	−0.81	NA
CL12067.Contig3_All	L-tryptophan---pyruvate aminotransferase [EC:2.6.1.99]	+ 1.23	NA
Unigene30442_All	prolyl 4-hydroxylase [EC:1.14.11.2]	−0.82	NA
CL14173.Contig1_All	prolyl 4-hydroxylase [EC:1.14.11.2]	−0.74	NA
CL2487.Contig3_All	anthranilate synthase component I [EC:4.1.3.27]	−0.81	NA
CL4443.Contig1_All	ornithine decarboxylase [EC:4.1.1.17]	+ 1.79	NA
CL6314.Contig4_All	aspartyl-tRNAsynthetase [EC:6.1.1.12]	+ 1.24	NA
CL11129.Contig1_All	alanyl-tRNAsynthetase [EC:6.1.1.7]	−0.69	NA
CL6813.Contig1_All	dihydropyrimidinase [EC:3.5.2.2]	1.22	NA
CL4336.Contig2_All	serine O-acetyltransferase [EC:2.3.1.30]	+ 1.6	NA
CL5396.Contig1_All	5-methylthioribose kinase [EC:2.7.1.100]	+ 1.39	NA
CL5396.Contig2_All	5-methylthioribose kinase [EC:2.7.1.100]	+ 1.39	NA
CL6794.Contig1_All	histidinol-phosphatase [EC:3.1.3.15]	+ 1.23	NA
CL1927.Contig4_All	Polyamine oxidase [EC: 1.5.3.14 1.5.3.16 1.5.3.-]	−0.79	NA
CL11987.Contig3_All	acetyl-CoA carboxylase[EC:6.4.1.2]	+ 1.34	NA
CL8633.Contig2_All	omega-hydroxypalmitate O-feruloyltransferase [EC:2.3.1.188]	+ 1.33	NA
CL8036.Contig2_All	fatty acid omega-hydroxylase [EC:1.14.-.-]	−0.5	NA
CL1854.Contig4_All	phosphoribosyl-AMP cyclohydrolase [EC 3.5.4.19]	− 0.69	NA
CL1854.Contig4_All	phosphoribosyl-ATP pyrophosphohydrolase [EC:3.6.1.31]	−0.69	NA
CL8479.Contig5_All	ATP phosphoribosyltransferase [EC:2.4.2.17]	−0.68	NA
CL6952.Contig1_All	alpha-galactosidase [EC:3.2.1.22]	+ 1.23	NA
CL5785.Contig5_All	glycerol kinase [EC:2.7.1.30]	+ 1.27	NA
CL7163.Contig2_All	phosphatidatecytidylyltransferase [EC:2.7.7.41]	+ 1.23	NA
Unigene6361_All	phosphatidylserine decarboxylase [EC:4.1.1.65]	+ 1.32	NA
CL12560.Contig3_All	glycerophosphoryldiesterphosphodiesterase [EC:3.1.4.46]	+ 1.8	NA
CL2234.Contig1_All	glucan endo-1,3-beta-glucosidase 1/2/3 [EC:3.2.1.39]	−0.71	NA
CL7420.Contig1_All	AMP deaminase [EC:3.5.4.6]	+ 1.25	NA
Unigene21084_All	nucleoside-diphosphate kinase [EC:2.7.4.6]	+ 1.21	NA
Unigene19886_All	lipoxygenase [EC:1.13.11.12]	+ 1.24	NA
Unigene19887_All	lipoxygenase [EC:1.13.11.12]	+ 1.51	NA
CL2477.Contig4_All	lipoxygenase [EC:1.13.11.12]	+ 1.38	NA
CL2477.Contig7_All	lipoxygenase [EC:1.13.11.12]	+ 1.64	NA
CL749.Contig1_All	alcohol dehydrogenase class-P [EC:1.1.1.1]	+ 1.28	NA
CL17848.Contig2_All	hexokinase [EC:2.7.1.1]	−0.8	NA
CL2098.Contig1_All	alpha-glucosidase [EC:3.2.1.20]	+ 1.46	NA
CL2521.Contig11_All	beta-fructofuranosidase [EC:3.2.1.26]	+ 0.75	NA
CL2521.Contig3_All	beta-fructofuranosidase [EC:3.2.1.26]	+ 1.3	NA
Unigene30358_All	beta-fructofuranosidase [EC:3.2.1.26]	+ 1.3	NA
Unigene7378_All	12-oxophytodienoic acid reductase [EC:1.3.1.42]	+ 1.29	NA
CL5130.Contig1_All	phospholipase A1 [EC:3.1.1.32]	−0.82	NA
Unigene556_All	12-oxophytodienoic acid reductase [EC:1.3.1.42]	−0.7	NA

The increased levels of histidinol-phosphatase (EC 3.1.3.15) and primary-amine oxidase (EC 1.4.3.21) corresponded with the observed lower histone and phenylalanine concentrations. Similarly, a high level of serine *O*-acetyltransferase (EC 2.3.1.30) also corresponded with patterns in cysteine and serine metabolism. In addition, high levels of uracil conversion to β-alanine might have been supported by enhanced activity of dihydropyrimidinase (EC 3.5.2.2).

Expression of L-tryptophan-pyruvate aminotransferase (EC 2.6.1.99) is responsible for the formation of tryptophan from indolepyruvate, which is then converted into anthranilate. The ATP and AMP are directly converted to ADP and IMP via upregulation of nucleoside-diphosphate kinase (EC 2.7.4.6) and AMP deaminase (EC 3.5.4.6), which could result in increased levels of xanthine, guanosine, and 2′, 3′-cyclic GMP. Interestingly, acetyl-CoA carboxylase (EC 6.4.1.2) plays a vital role in increasing or reducing concentrations of hexadecenoic acid, capric acid, and palmitic acid. In resistant genotype, galactosylglycerol was significantly accumulated because of increased concentrations of glycerol kinase (EC 2.7.1.30) and reductions in the hexokinase pool.

In addition, the higher metabolic levels of alpha-galactosidase (EC 3.2.1.22), alpha-glucosidase (EC 3.2.1.20), and beta-fructofuranosidase (EC 3.2.1.26) indicated stronger metabolism of galactose. Moreover, colnelenic acid, traumatic acid, stearidonic acid, alpha-linolenic acid, and 13(S)-HPOT (9Z,11E,15Z)-(13S)-13-Hydroperoxyoctadeca-9,11,15-trienoic acid) are all involved in alpha-linolenic acid metabolism, in which genes encoding enzymes, such as phospholipase A1 (EC 3.1.1.32), lipoxygenase (EC 1.13.11.12), and alcohol dehydrogenase class-P (EC 1.1.1.1) were each either strongly downregulated or upregulated.

## Discussion

Plants subjected to water stress undergo a range of physiological changes [30]. In our previous study, in comparison to susceptible genotype, a higher level of CAT activity was exhibited in resistant genotype against short-term drought; however, SOD and MDA activity showed no significant differences between the two *L. multiflorum* genotypes when treated with 1 h of drought [20]. In contrast, in the present study, a significant difference was noted between the two annual ryegrass genotypes in response to long-term drought (Fig. 1). As the number of days of drought increased, tolerant plants exhibited higher levels of CAT and SOD activity, higher RWC and chlorophyll content, and lower MDA content (Fig. 1). Research conducted by Mastalerczuk et al. [31] and Borawska-Jarmujłowicz et al. [32] on perennial ryegrass showed that significant reductions in chlorophyll contents were observed after subjecting seedlings to abiotic stresses. These findings are consistent with those of the present study. Thus, the activity of antioxidant enzymes and changes in chlorophyll content play important roles in increasing the tolerance of resistant genotype to drought stress.

Alterations in membrane lipid composition represent an important response to environmental stresses [33, 34]. Drought stress reduces the accumulation of fatty acids, such as palmitic acid, hexadecenoic acid, picolinic acid, and stearidonic acid [4, 35, 36], while substantially increasing the levels of most fatty acids, with the exception of pimelic acid, capric acid, (2’E,4’Z,7’Z,8E)-colnelenic acid, alpha-linolenic acid, traumatic acid (TA) and acetyl-CoA carboxylase (ACCase) [37]. Tayeh et al. [38] showed that phospholipase A1 (PLA1) in plants plays important roles in the hydrolysis of phospholipids during cold stress. Andrade et al. [39] found that lipoxygenase (LOX) is involved in defense against drought stress in rice and sugarcane transgenic plants. Uma and Podile [40] reported that the transcripts encoding 9-LOX and a 9-LOX-derived compound, colnelenic acid, are significantly upregulated in resistant host tomato plants. Similarly, Cao et al. [41] reported that levels of alpha-linolenic acid and colnelenic acid were increased following treatment with exogenous methyl jasmonate (MeJA) . In the present study, phospholipase A1 enhanced the production of alpha-linolenic acid, which is converted to colnelenic acid; an effect that was directly induced by lipoxygenase in the tolerant annual ryegrass subjected to drought conditions (Fig. 6). These results showed that alpha-linolenic acid and colnelenic acid are involved in the regulation of membrane composition in drought-tolerant genotype.

In addition, TA is a plant wound hormone that can eliminate the negative effects of salt stress and oxidative stress in *Chlorella vulgaris* [42, 43]. Consistent with our previous results, a high TA content was detected in the resistant plants, indicating that the metabolite contributes to enhanced stress tolerance in drought-treated annual ryegrass. Moreover, levels of acetyl-CoA carboxylase (ACCase) and omega-hydroxypalmitate *O*-feruloyl transferase in the annual ryegrass plants were enhanced by drought treatment. We found that reductions in the levels of palmitic acid and hexadecenoic acid were controlled by ACCase. The changes observed in ACCase and palmitic acid in lipid metabolism were consistent with the results of Kwan et al. [44].

Most free amino acids accumulate under drought stress [45], and this is thought to be an important adaptation [46, 47]. In the present study, downregulated branched-chain aminotransferase (BCAT) directly reduced L-valine and L-leucine levels. Anthranilate synthase (AS) causes a leucine-to-proline change, which leads to a reduction in the proline content of annual ryegrass. In addition, the upregulation of histidinol-phosphatase is involved in L-histidine degradation. Furthermore, tyrosine amino transferase and phenylpyruvate tautomerase might be jointly involved in the degradation of phenylalanine. This finding has not been observed in previous studies.

The response of 5-methylthioribose to drought stress in drought-tolerant annual ryegrass is induced by cysteine and methionine metabolism, which was controlled in the present study by the two downregulated enzymes, tyrosine aminotransferase and 5-methylthioribose kinase. Moreover, this compound has not been associated with drought tolerance in previous studies. Anthranilate acts as a precursor for tryptophan biosynthesis and reduces indoleacetic acid (IAA) synthesis [48]. Anthranilate formation resulted in indirect induction of IAA production, a novel finding in annual ryegrass that was consistent with the results of Hartmann and Zimmer [49].

The behavior of 2′,3′-cyclic nucleotide variants has been characterized in animal tissues, but not in plant materials [50]. According to our research, only 2′,3′-cyclic GMP was detected in annual ryegrass, which is involved in guanine production. Glucosylglycerol, found in many cyanobacteria, and galactosylglycerols (floridoside and isofloridoside) accumulate in eukaryotic algae under salt stress conditions [51]. In the present study, galactosylglycerol production was involved in glycolysis and fructose/mannose metabolism in response to drought, indicating that the concentration of galactosylglycerol plays a role in drought tolerance of annual ryegrass. In another study, galactose levels in drought-resistant potato genotypes were observed to be much higher than those in corresponding controls [52], a finding that is consistent with our results.

Transcription factors involved in transcriptional and post-transcriptional mechanisms regulate the accumulation of various metabolites by activating the expression of biosynthetic enzymes. Based on our omics data, various TF families, including MYB, bZIP, and bHLH, might regulate core metabolic processes during drought stress. The R2R3-MYB transcription factor AtMYB41 is induced in response to desiccation, and activates cuticle biosynthesis In *Arabidopsis thaliana* under biotic stress conditions [53]. In addition, AtMYB96 positively regulates the expression of lipid-transfer protein 3 (*LTP3*) via direct binding to the *LTP3* promoter, and thereby enhances plant drought stress tolerance [54].

In response to drought stress, many plant species accumulate high levels of compatible osmolytes, such as Pro (proline), Gly betaine, or sugar alcohols, which are thought to be associated with stress adaptation. In higher plants, L-Pro is synthesized from L-Glu via delta-1-pyrroline-5-carboxylate (P5C) by two enzymes, P5C synthetase (P5CS) and P5C reductase (P5CR) under drought stress. The L-Pro is metabolized to L-Glu via P5C by two enzymes, Pro dehydrogenase (ProDH) and P5C dehydrogenase, during recovery from stress [55, 56].

Satoh and colleagues [57] first identified the group of S bZIP TFs, such as AtbZIP11, AtbZIP44, AtbZIP2, and AtbZIP53, which become involved in Pro metabolism by binding to the *cis*-acting element ACTCAT in the *ProDH* promoter. Later, Hanson and colleagues [58] reported that AtbZIP11 could affect amino acid metabolism by regulating the expression of *ASPARAGINE SYNTHETASE1* and *ProDH2* during low energy stress. Post-transcriptional regulatory mechanisms are also very important for drought stress tolerance in plants. It has been reported that AtbZIP1 and AtbZIP53 can be specifically heterodimerized with group C bZIP TFs to bind directly to the promoters of *ASPARAGINE SYNTHETASE1* and *ProDH2* [59]*.* In addition, the *Arabidopsis* type-B response regulator 18 (ARR18) physically interacts with AtbZIP63; an interaction that negatively interferes with the transcriptional activity of AtbZIP63 on the *ProDH1* promoter under osmotic stress [60]. Recently, the key TF AtbHLH112 involved in Pro metabolism was identified by Liu et al. [61]. AtbHLH112 could activate the expression of *P5CS* and repress the expression of *P5CDH* and *ProDH* to increase Pro levels under abiotic stress tolerance. Taken together, the omics data suggest that post-transcriptional and transcriptional regulation of TFs that affect proteins and enzymes could mediate lipid and amino acid metabolism, thereby promoting adaptation to drought stress in annual ryegrass*.*

Over the last few decades, extensive efforts have been made to produce drought-tolerant ryegrass genotypes using molecular and biotechnological methods, such as the production of genetically modified or transgenic plants, with a focus on lipid and amino acid metabolism [62, 63]. Transgenic expression of some drought stress-related genes involved in lipid and amino acid metabolism could provide a more rapid strategy to achieve improved drought stress tolerance in ryegrass.

## Conclusions

In order to explore the molecular mechanism associated with drought tolerance in two annual ryegrass genotypes, we identified differentially expressed metabolites and their corresponding proteins and transcripts that are involved in 23 core metabolic processes, in response to short-term drought stress. The regulatory networks were inferred using MCoA and correlation analysis to reveal the relationships among the expression of transcripts, proteins, and metabolites that highlight the corresponding elements of these core metabolic pathways. This study provides useful insights into the molecular mechanisms of drought resistance and represents a promising approach toward enhancing drought tolerance in forage grass.

## Additional files

Additional file 1: Figure S1. Sampling strategy for annual ryegrass under drought treatment for transcriptome, proteome, and metabolome analyses. (TIFF 2865 kb)

Additional file 2: Table S1. Detailed information of genes used for real-time-PCR analysis (DOCX 15 kb)

Additional file 3: Figure S2. Hierarchical clustering of different types of compounds detected in two *L. multiflorum* genotypes in the positive mode (A) and negative mode (B). (TIFF 1337 kb)

Additional file 4: Figure S3. Heatmaps of metabolite–metabolite correlations in positive mode (A) and negative mode (B). Metabolites were grouped by compound class, and each square represents the correlation between the metabolites indicated in the column and row headings. (TIFF 2818 kb)

Additional file 5: Figure S4. Summary of annotated unigenes identified using different functional databases (A) and unigenes that encode transcription factors (TFs) classified into TF families (B). (TIFF 1059 kb)

Additional file 6: Figure S5. Volcano plot of DEGs induced by drought treatment in two *L. multiflorum* genotypes at four time points. (TIFF 992 kb)

Additional file 7: Figure S6. The real-time PCR confirmation of RNA-Seq data in the drought-resistant (A) and drought-susceptible (B) annual ryegrass; Western blot validation of iTRAQ results in two *L. multiflorum* genotypes (C). (TIFF 2571 kb)

## Abbreviations

ACT: Acetonitrile
ANOVA: Analysis of variance
APX: Ascorbic acid peroxidase
AS: Anthranilate synthase
CAT: Catalase
COG: Clusters of orthologous groups
DEG: Differentially expressed genes
DEM: Differentially expressed metabolites
DEP: differentially expressed proteins
FA: Formic acid
FDR: False discovery rate
FPKM: Fragments per kilobase of transcript sequence per million base pairs
GO: Gene Ontology
GSEA: Gene set enrichment analysis
IAA: Indoleacetic acid
IMP: Inosine monophosphate
KEGG: Kyoto Encyclopedia of Genes and Genomes
LOX: Lipoxygenase
LSD: Least significant difference
MCoA: Multiple co-inertia analysis
MDA: Malondialdehyde
MeJA: Methyl jasmonate
MGF: Mascot generic format
OPLS-DA: Orthogonal projections to latent structures discriminant analysis
PCA: Principal component analyses
PLS-DA: Partial least squares discriminant analysis
PVDF: Polyvinylidene fluoride
REC: Relative electrical conductivity
RP: Resolving power
RSR: Root-to-shoot ratio
RT: Retention time
RWC: Relative water content
SH: Seedling height
SOD: Superoxide dismutase
TA: Traumatic acid
TCA: Tricarboxylic acid
TF: Transcription factors
VIP: Variable importance in the project
