# Supplementary figures and images for: Integrated omics data of two annual ryegrass (Lolium multiflorum L.) genotypes reveals core metabolic processes under drought stress
Source: BMC Plant Biol. 2018 Jan 30;18:26. doi: 10.1186/s12870-018-1239-z (PMC5789592; doi:10.1186/s12870-018-1239-z)

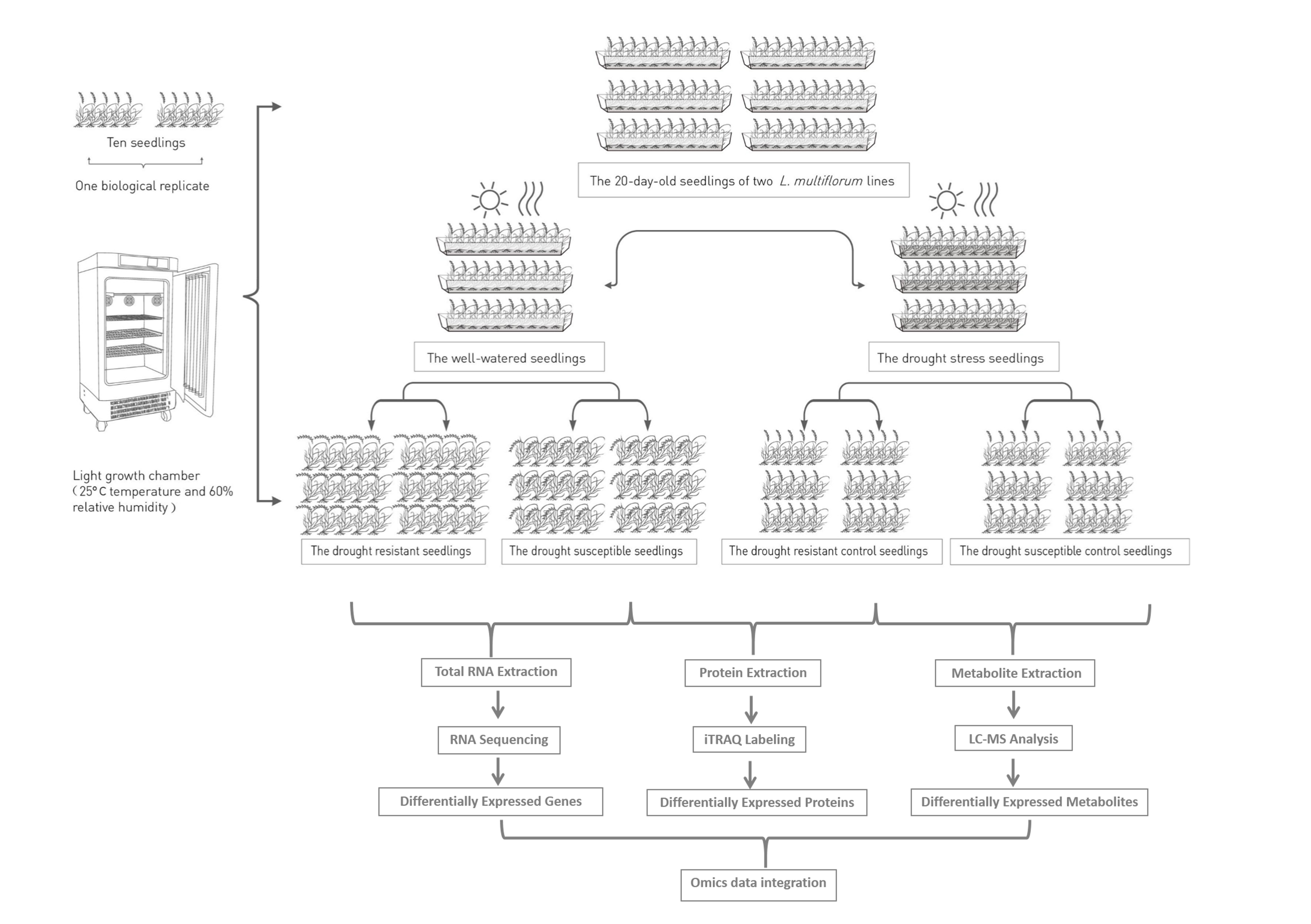

Supplement: Supplementary file 1 — Sampling strategy for annual ryegrass under drought treatment for transcriptome, proteome, and metabolome analyses. (TIFF 2865 kb) [file 12870_2018_1239_MOESM1_ESM.tif]

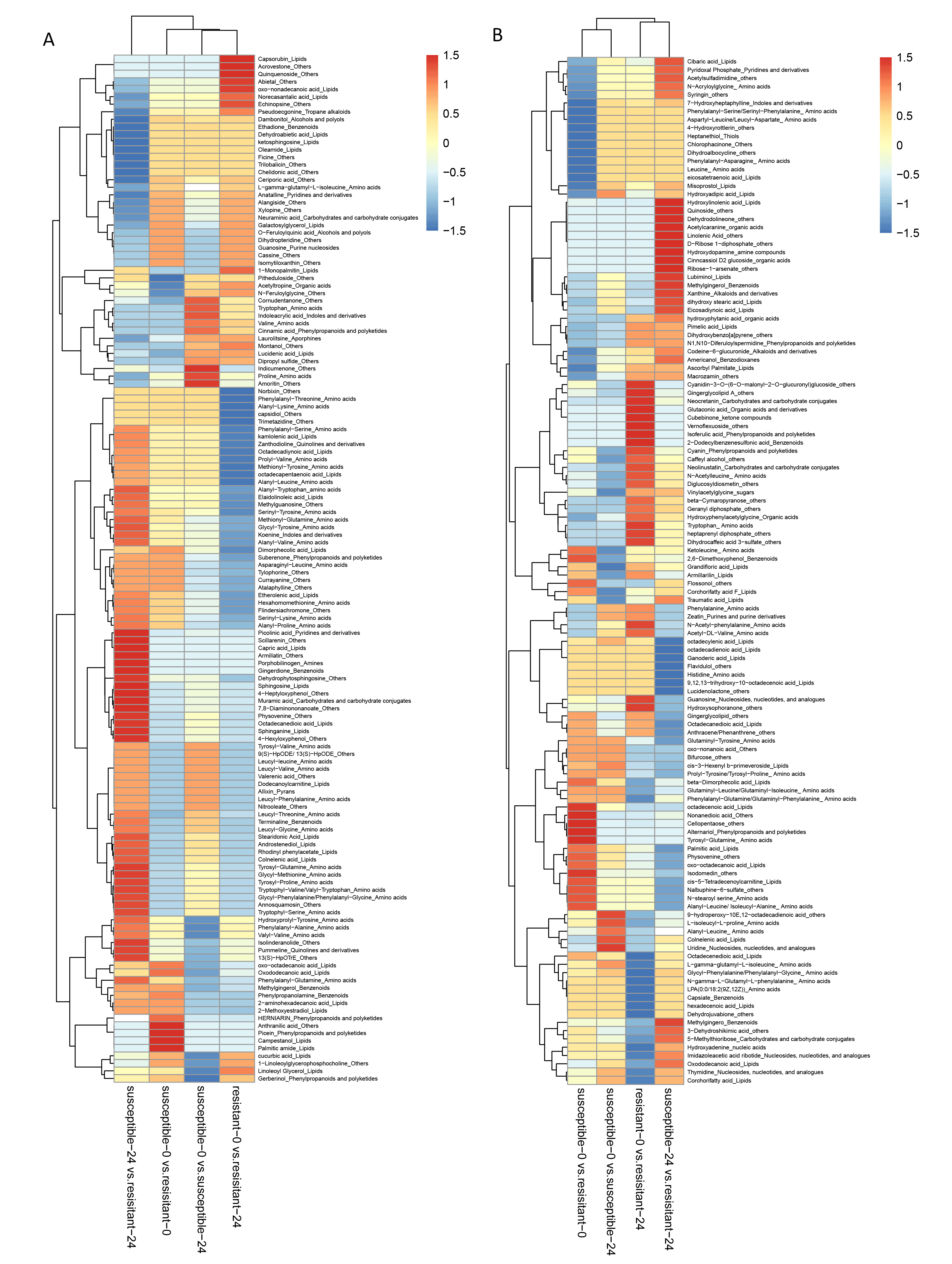

Supplement: Supplementary file 3 — Hierarchical clustering of different types of compounds detected in two L. multiflorum genotypes in the positive mode (A) and negative mode (B). (TIFF 1337 kb) [file 12870_2018_1239_MOESM3_ESM.tif]

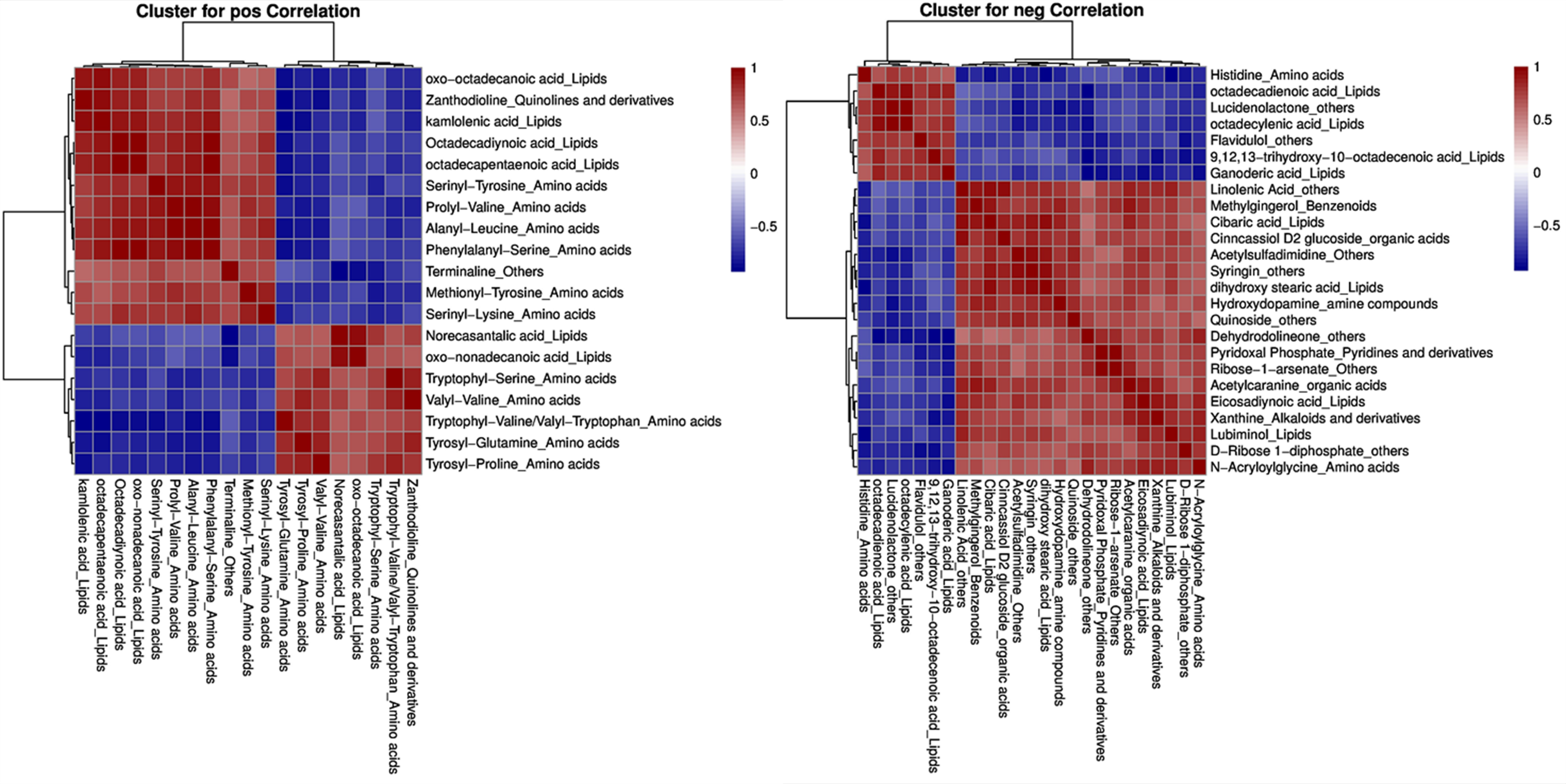

Supplement: Supplementary file 4 — Heatmaps of metabolite–metabolite correlations in positive mode (A) and negative mode (B). Metabolites were grouped by compound class, and each square represents the correlation between the metabolites indicated in the column and row headings. (TIFF 2818 kb) [file 12870_2018_1239_MOESM4_ESM.tif]

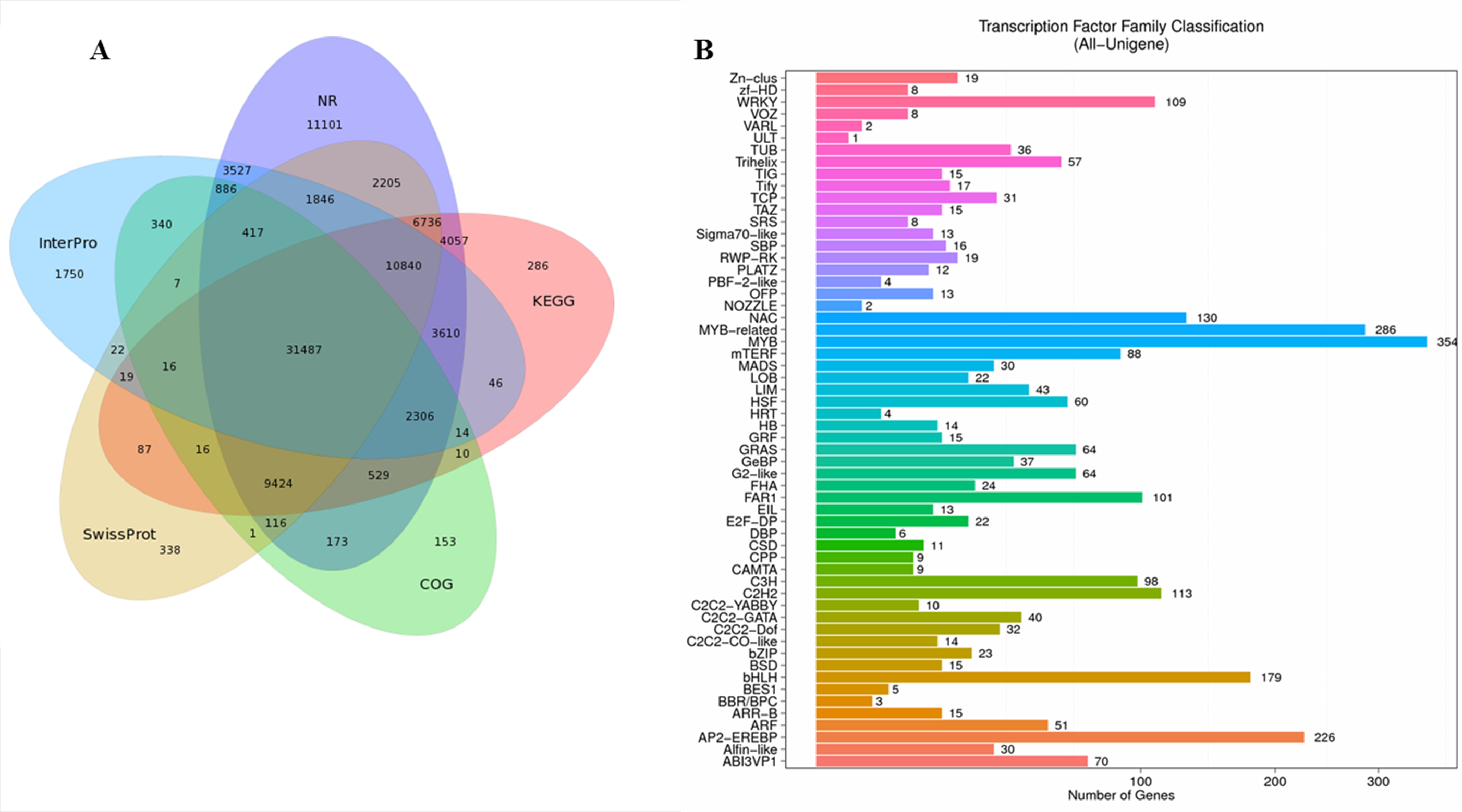

Supplement: Supplementary file 5 — Summary of annotated unigenes identified using different functional databases (A) and unigenes that encode transcription factors (TFs) classified into TF families (B). (TIFF 1059 kb) [file 12870_2018_1239_MOESM5_ESM.tif]

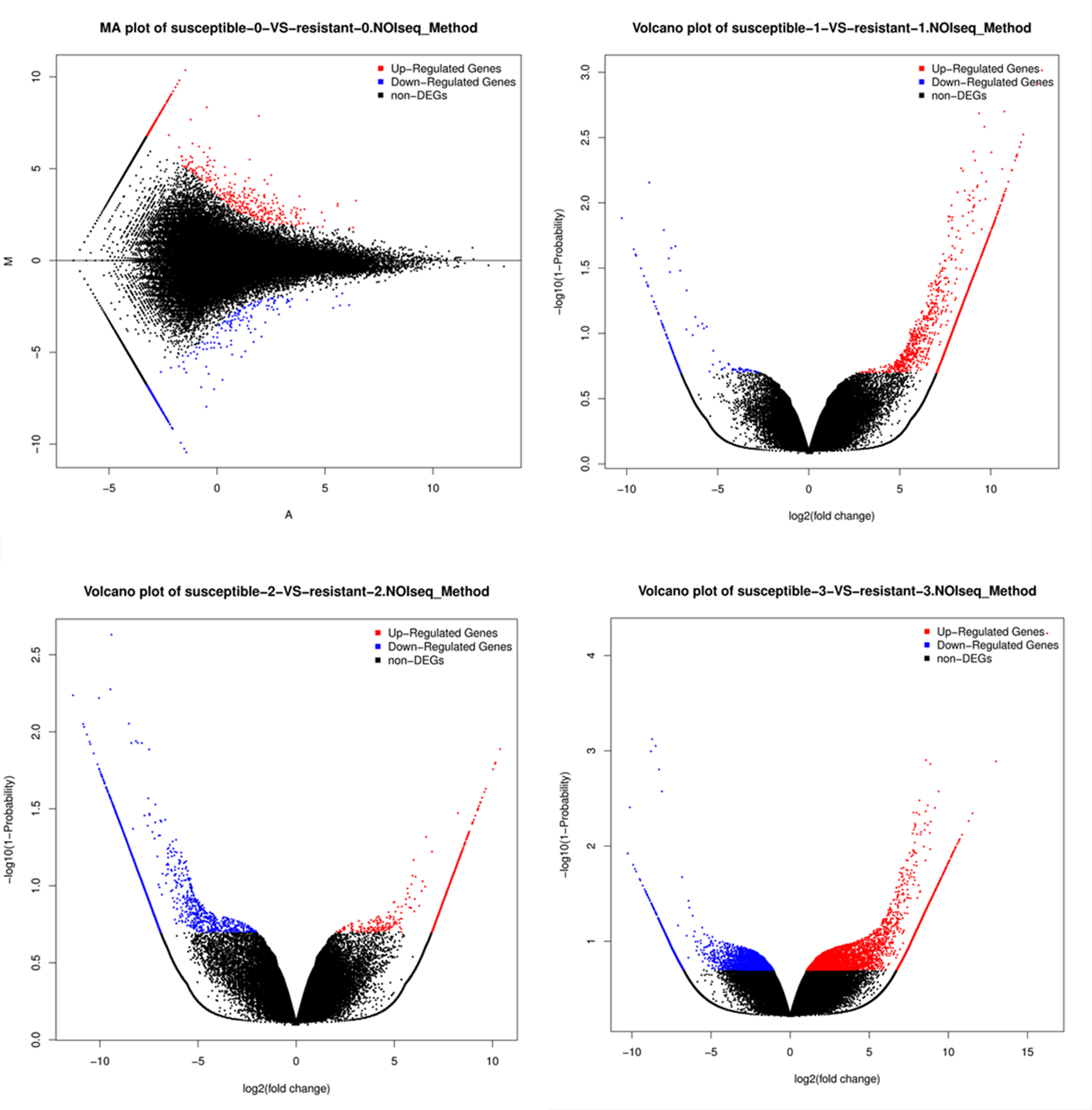

Supplement: Supplementary file 6 — Volcano plot of DEGs induced by drought treatment in two L. multiflorum genotypes at four time points. (TIFF 992 kb) [file 12870_2018_1239_MOESM6_ESM.tif]

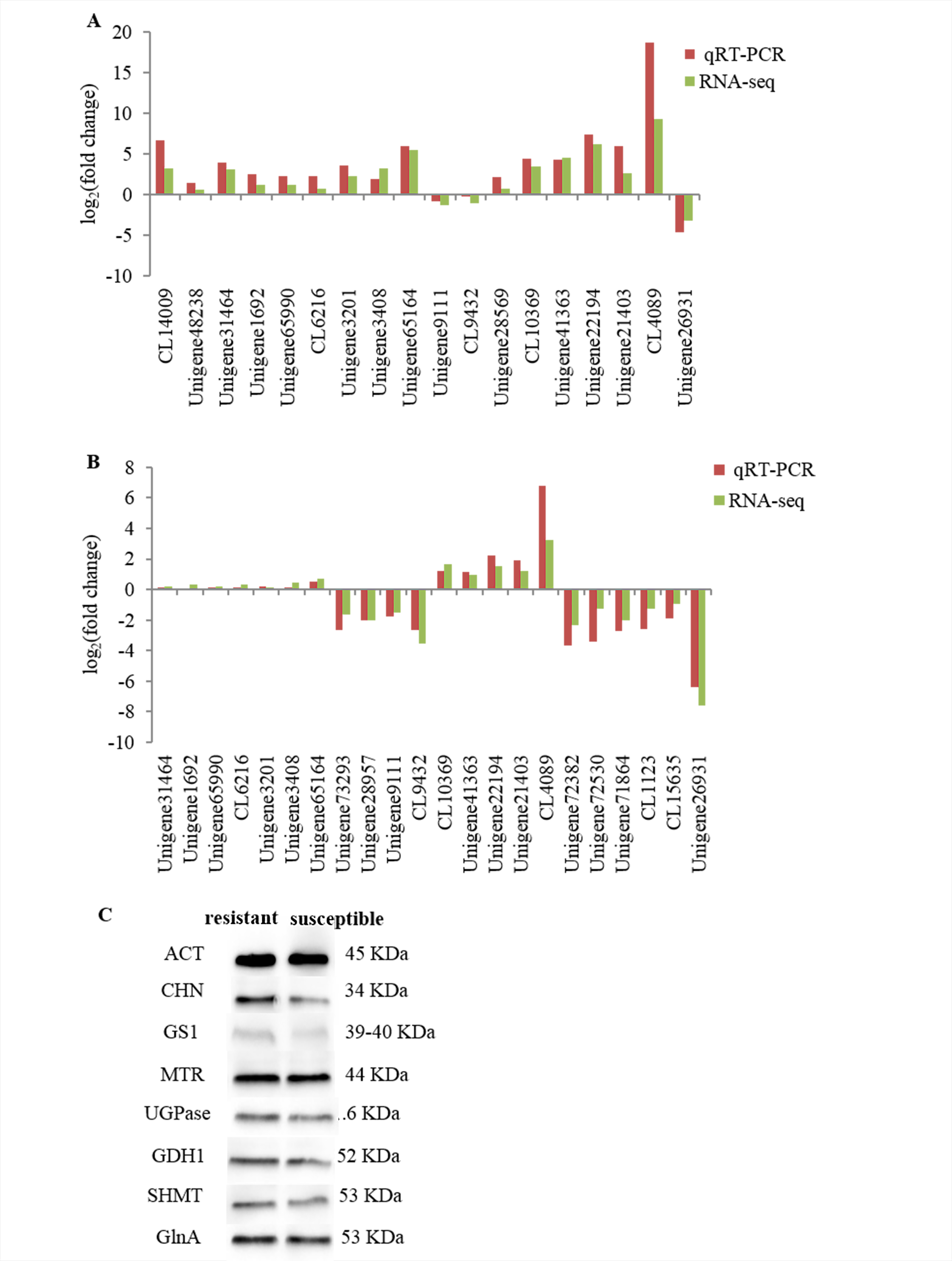

Supplement: Supplementary file 7 — The real-time PCR confirmation of RNA-Seq data in the drought-resistant (A) and drought-susceptible (B) annual ryegrass; Western blot validation of iTRAQ results in two L. multiflorum genotypes (C). (TIFF 2571 kb) [file 12870_2018_1239_MOESM7_ESM.tif]
