# Supplementary material for: Integrated omics data of two annual ryegrass (Lolium multiflorum L.) genotypes reveals core metabolic processes under drought stress
Source: BMC Plant Biol. 2018 Jan 30;18:26. doi: 10.1186/s12870-018-1239-z (PMC5789592; doi:10.1186/s12870-018-1239-z)
Supplement: Supplementary file 2 — Detailed information of genes used for real-time-PCR analysis (DOCX 15 kb) [file 12870_2018_1239_MOESM2_ESM.docx]

Supplemental Table 1.Detailed information of genes used for real-time-PCR analysis

| **Seq-ID** | **Gene name** | **Primer Sequence (5'-3') and Tm(**℃**)** | **GO-CellularComponent** |
| --- | --- | --- | --- |
| AJ585201 | Actin | F:TCCTCACGCCATTCTT (59.10)  R: TCTCCTTGATGTCCCT (59.08) | GO:0008372; cellular component; |
| CL14009 | 12-oxophytodienoic acid reductase  [EC:1.3.1.42] | F: TCGTAATCAAAGCTCGGTTG (58.92)  R: GAGCAGCTGAAATGGTGAAA (59.00) | －－ |
| Unigene48238 | formate dehydrogenase [EC:1.2.1.2] | F: GAAGAGGTATGCTGATGGCA (58.83)  R: AGACCATACGCCTTGGTAGC (59.22) | GO:0005739//mitochondrion;GO:0009579//thylakoid;GO:0009507//chloroplast;GO:0016020//membrane; |
| Unigene31464 | aspartylaminopeptidase [EC:3.4.11.21] | F: TCGTAATCAAAGCTCGGTTG (58.92)  R: GAGCAGCTGAAATGGTGAAA (59.00) | GO:0005829//cytosol;GO:0005774//vacuolarmembrane;GO:0009570//chloroplast stroma |
| Unigene1692 | 3-hydroxyacyl-CoA dehydrogenase [EC: 1.1.1.211 | F: GGTAAGACTCCCGTCGTTGT (59.06)  R: GTATGGGAAGAAGGCACGAT (59.01) | GO:0005829//cytosol;GO:0005618//  cell wall;GO:0005777//peroxisome;GO:0005730//nucleolus; |
| Unigene65990 | peroxiredoxin [EC:1.11.1.15] | F: GCCGATCCAACTCATACCTT (59.01)  R: TGCATGCTTGAGGATACCAT (59.10) | GO:0009941//chloroplastenvelope;  GO:0048046//apoplast;GO:0010319//stromule;GO:0005829//cytosol; |
| CL6216 | enoyl-CoA hydratase [EC:4.2.1.17] | F: ACACAAGCAAAGACAATGCC (58.78)  R: AAGCCCTGATCACCACATTT (59.41) | GO:0005856//cytoskeleton;GO:0005777//peroxisome |
| Unigene3201 | glycerol kinase [EC:2.7.1.30] | F: CTCGTAACACCGACACCATC (59.01)  R: CGTTGTCTAACATCCATCGG (59.00) | GO:0005829//cytosol |
| Unigene3408 | 4,4-dimethyl-9beta,19-cyclopropylsterol-4alpha-methyl oxidase [EC:1.14.13.72] | F: TACGCCGATTTCATTTGTGT (59.04)  R: GCATCGATCACAAATCTTGC (59.23) | GO:0005768//endosome;GO:0005802//trans-Golginetwork;GO:0016020//  membrane;GO:0005794//Golgi apparatus |
| Unigene65164 | 12-oxophytodienoic acid reductase [EC:1.3.1.42] | F: GCTTGTCGGTACCGTAGGTT (59.12)  R: CCAGGGTTCTTCTGGAACAT (58.99) | －－ |
| Unigene73293 | NADH dehydrogenase [EC:1.6.5.3 1.6.99.3] | F: CACCACAGACTTGGGACTTG (59.15)  R: ATGCTGAATTCCTCCTCCAC (59.09) | GO:0005759//mitochondrial matrix |
| Unigene28957 | long-chain acyl-CoA synthetase [EC:6.2.1.3] | F: ACGACCAAAGTGGTGAACAA (59.03)  R: TGCCGAAATCAAGACACAAT (59.13) | GO:0009941//chloroplastenvelope;  GO:0005794//Golgi apparatus;  GO:0005783//endoplasmic reticulum |
| Unigene9111 | long-chain acyl-CoA synthetase [EC:6.2.1.3] | F: CACTGTTGGTCCACCTGTTC (58.99)  R:CATCCAAACCTCACCTTGTG (58.98) | GO:0005777//peroxisome;GO:0016020//membrane |
| CL9432 | ubiquitin-conjugating enzyme E2 D/E [EC:6.3.2.19] | F: CCCAAAGATCAACTTCGTCA (58.96)  R:GGGTCAGTCAACAAAGAACTG(59.01) | GO:0005737//cytoplasm;GO:0005634//nucleus;GO:0005886//plasma membrane |
| Unigene28569 | 6-phosphogluconate dehydrogenase [EC:1.1.1.44] | F: ACATTAGCTATCGGCAAGGG (59.20)  R: CGAAGAAATCACGTTGTGCT (58.93) | GO:0005829//cytosol;GO:0005777//peroxisome;GO:0009570//chloroplast stroma |
| CL10369 | branched-chain amino acid aminotransferase [EC:2.6.1.42] | F: CTCGATGAGGATGATTGGAA (58.59)  R: ACCGGGACTGGAGAAGAGA (59.78) | GO:0009570//chloroplast stroma;  GO:0005829//cytosol |
| Unigene41363 | tyrosine decarboxylase [EC:4.1.1.25] | F: CGACTACAAGGACTGGCAGA (59.03)  R:CAATACCACCCACAGCTTCA (59.57) | －－ |
| Unigene22194 | solute carrier family 32 | F: TCGTTCATCTCATCGGTAGC (58.83)  R: TTCACCAGCAATGACTCCAT (59.09) | GO:0016021//integral to membrane;  GO:0005886//plasma membrane |
| Unigene21403 | aspartate aminotransferase and glutamate [EC:2.6.1.1] | F: GGGATGCATTTGGAGATGA (59.39)  R: TGAGCAGAGTCGTTGCTTCT (58.91) | GO:0009507//chloroplast |
| CL4089 | histidine decarboxylase [EC:4.1.1.22] | F: GATGCAACGATCATGGGTAG (58.95)  R: TGCGAGATAATGAGCGTTTC (59.02) | GO:0005886//plasma membrane;GO:0005829//cytosol |
| Unigene72382 | glyceraldehyde 3-phosphate dehydrogenase [EC:1.2.1.12] | F: GGACCAGGTTGTGTCAACAG (58.99)  R: CCACTCTTGTGGAATAGCCA (58.72) | GO:0016020//membrane |
| Unigene72530 | glutamate decarboxylase [EC:4.1.1.15] | F: ATCATCTTGGCTACCTTGGC (59.15)  R: TCGAGATAGCGGATAGCCTT (59.05) | GO:0009506//plasmodesma;GO:0005634//nucleus;GO:0005829//cytosol |
| Unigene71864 | glycine hydroxymethyltransferase [EC:2.1.2.1] | F: CAAGTGGCTGAGTTCCTTGA(59.01)  R: AAGGCCTTGAGCTCATCATT (58.89) | --  GO:0005886//plasmamembrane;GO:0009507//chloroplast; |
| CL1123 | glyceraldehyde 3-phosphate dehydrogenase [EC:1.2.1.12] | F: TGGGTGAGCGTAATTCTTCA (59.27)  R: CGTTATCGTACCAGGCAATG (59.06) | GO:0048046//apoplast;GO:0005618//cellwall; GO:0005740//mitochondrialenvelope; GO:0005730//nucleolus; |
| CL15635 | cinnamyl-alcohol dehydrogenase [EC:1.1.1.195] | F: TCAACCTGGTCAGAAAGTCG (58.85)  R: AAACGCGTACACTTCAGCAC (59.00) | -- |
| Unigene26931 | glyceraldehyde 3-phosphate dehydrogenase [EC:1.2.1.12] | F: CTGTCGGTTCCGTTATTCCT (59.05)  R: TGAAGCACCTTTCTCAATGC (59.00) | GO:0009536//plastid;GO:0016020//membrane |
